# Non-Terrestrial Networks for Energy-Efficient Connectivity of Remote IoT Devices in the 6G Era: A Survey

**DOI:** 10.3390/s24041227

**Published:** 2024-02-15

**Authors:** Stefanos Plastras, Dimitrios Tsoumatidis, Dimitrios N. Skoutas, Angelos Rouskas, Georgios Kormentzas, Charalabos Skianis

**Affiliations:** 1 Department of Information and Communication Systems Engineering, University of the Aegean, 83200 Samos, Greece; d.tsoumatidis@aegean.gr (D.T.); d.skoutas@aegean.gr (D.N.S.); gkorm@aegean.gr (G.K.); cskianis@aegean.gr (C.S.); 2 Department of Digital Systems, University of Piraeus, 18532 Piraeus, Greece; arouskas@unipi.gr

**Keywords:** Internet of Things (IoT), 5G, 6G, Internet of Remote Things (IoRT), unmanned aerial vehicles (UAVs), satellites, non terrestrial networks (NTNs)

## Abstract

The Internet of Things (IoT) is gaining popularity and market share, driven by its ability to connect devices and systems that were previously siloed, enabling new applications and services in a cost-efficient manner. Thus, the IoT fuels societal transformation and enables groundbreaking innovations like autonomous transport, robotic assistance, and remote healthcare solutions. However, when considering the Internet of Remote Things (IoRT), which refers to the expansion of IoT in remote and geographically isolated areas where neither terrestrial nor cellular networks are available, internet connectivity becomes a challenging issue. Non-Terrestrial Networks (NTNs) are increasingly gaining popularity as a solution to provide connectivity in remote areas due to the growing integration of satellites and Unmanned Aerial Vehicles (UAVs) with cellular networks. In this survey, we provide the technological framework for NTNs and Remote IoT, followed by a classification of the most recent scientific research on NTN-based IoRT systems. Therefore, we provide a comprehensive overview of the current state of research in IoRT and identify emerging research areas with high potential. In conclusion, we present and discuss 3GPP’s roadmap for NTN standardization, which aims to establish an energy-efficient IoRT environment in the 6G era.

## 1. Introduction

The primary goal of the Internet of Things (IoT) is to create a comprehensive computing environment where everyday objects can sense and interact with their surroundings, bridging the physical and digital realms [1]. The IoT is expected to continue growing further, with forecasts of over 5.5 billion cellular IoT connections by 2027, reflecting its fast expansion [2]. The rapid development of IoT technology opens up numerous applications that have the potential to transform our daily lives [3,4]. These IoT technologies are being leveraged to enhance various aspects of our environment, giving rise to concepts like smart buildings, smart cities, flexible energy infrastructure, and smart agriculture, effectively embedding intelligence within these domains [5,6]. In the realm of smart agriculture, for example, the activation or deactivation of an irrigation system can be precisely controlled based on real-time data collected from humidity sensors deployed in remote fields. This automated approach not only optimizes irrigation efficiency but also enables remote monitoring and management of agricultural operations, while simultaneously providing valuable insights for comprehensive evaluation and analysis [7].

Efficient data transmission is paramount in the expansive realm of the IoT, as it underpins the seamless operation of IoT systems across various domains [8]. The continuous exchange of data and information between devices, data storage systems, and end-users is crucial for the effective functioning of IoT applications. Current IoT architectures employ a diverse range of communication technologies, encompassing short-range solutions tailored for localized environments like homes and industrial settings as well as long-range solutions capable of covering vast geographic areas [9,10]. The introduction of 5G technology marks a pivotal moment in the IoT landscape, addressing the evolving connectivity needs of IoT applications. The standardization of 4G-IoT systems, such as Long-Term Evolution Machine type communication (LTE-M) and NarrowBand Internet of Things (NB-IoT), continued to evolve in 5G standards [11,12].

These advancements collectively contribute to a transformative 5G-IoT ecosystem, unlocking a vast spectrum of possibilities for the future of connected devices and services [13,14,15,16]. The IoT is rapidly evolving, paving the way for innovative scenarios and expanding the range of existing IoT applications, laying the foundation for future 6G-IoT systems [17,18]. However, despite the progress made, there are still unique challenges that need to be addressed, particularly in the research area of the Internet of Remote Things (IoRT).

### Internet of Remote Things

The Internet of Remote Things (IoRT) extends the reach of the Internet of Things to remote and physically isolated regions where terrestrial and cellular networks are absent. This innovative paradigm broadens the scope of the IoT by connecting the farthest and the most remote corners of the world. However, IoRT deployments often encounter significant challenges, such as limited connectivity and harsh environmental conditions [18,19].

Despite these difficulties, there is a growing demand for IoRT applications, as they hold immense potential in various domains, including environmental monitoring, wildlife tracking, disaster relief management, and the delivery of critical healthcare and education services to under-served rural communities [20]. Thus, a number of specialized IoRT environments are formed, as illustrated in Table 1. The Internet of Agricultural Things (IoAT), for instance, has the potential to enhance agricultural productivity and income generation in remote places with arable land [7,21,22]. In oceans and seas, the Internet of Maritime Things (IoMT) is also an excellent example of an IoRT environment [23,24] where, for example, sensors on ships can transmit vital data, facilitating weather forecasts and enhancing maritime safety [25]. Specialized IoRT approaches are also required for the Internet of Underground Things (IoUT), which is applicable to subterranean operations such as mining [26,27,28], and the Internet of Underwater Things (IoUwT), which is applicable to marine exploration or aquaculture [29,30,31,32].

In addition, the Internet of Space, which extends IoT capabilities to satellites and extraterrestrial expeditions, is another prominent example of IoRT deployment [33,34]. In the Internet of Battle Things (IoBT), IoT-enabled devices on military assets, equipment, and personnel provide vital information, optimize deployment strategies, and improve situational awareness, thereby enhancing the efficiency and safety of military operations [35,36,37] even in the most remote areas.

Similarly, IoRT technologies are essential for gathering data on wildlife behavior, climate, and glacier melt in the far-flung and harsh Arctic, forming the Internet of the Arctic. These IoT systems enable scientists and researchers to study and comprehend the swiftly transforming Arctic conditions, which is essential for environmental monitoring and the development of strategies to mitigate the effects of climate change [38]. Hence, IoRT systems are essential for bridging isolated ecosystems in remote environments, enabling communication, data sharing, and intelligent operations.

This survey investigates the application of non-terrestrial networks to connect remote IoT systems within the framework of the Internet of Remote Things. The primary objectives are to identify the scope of Non-Terrestrial Networks (NTNs), classify technological and energy efficiency challenges, and discuss open issues towards 6G-IoT. Hence, the main contributions of the present study are:

- A thorough analysis of various NTN architectures, including satellite networks operating in different orbits, UAV-based networks, and hybrid NTNs, that can address the limitations of current terrestrial networks. Each NTN type is discussed in detail, outlining its strengths, limitations, and potential applications.
- Identifies and discusses key challenges in integrating NTNs into the IoRT ecosystem. It further outlines seven important research objectives for NTN integration in IoRT, ranging from energy efficiency and cost-effective deployment to enhanced coverage, throughput, data transmission reliability, and timely data acquisition.
- Delving into the research field of NTN-based IoRT, this study dissects three key approaches: satellite, hybrid satellite-UAV, and UAV-based systems. By further analyzing each category through the lens of individual research objectives, it unveils a comprehensive overview of this dynamic landscape.
- Exploring the future of IoRT, this study examines research trends and challenges while also spotlighting promising technologies. Focusing on the 3GPP (3rd Generation Partnership Project) standardization roadmap, it highlights the crucial role of upcoming research and standardization efforts in bridging remote connectivity gaps and paving the way for energy-efficient IoRT environments in the 6G era.

## 2. Related Work and Survey Scope

In order to establish clear research objectives for the present study, we conducted a comprehensive review of previous studies in the relevant research literature. Below, we present our key findings that emerged from this review, which are also summarized in Table 2.

The study in [39] provides a comprehensive overview of satellite communication systems for the IoT. However, it overlooks two crucial aspects: energy efficiency considerations in NTNs and the integration of Unmanned Aerial Vehicles (UAVs) in the IoRT framework, which are essential for the successful implementation of IoRT solutions. Similarly, while [40] while provides a comprehensive overview of the Low Earth Orbit (LEO) satellite-based IoRT architecture, the authors do not examine energy efficiency issues or the employment of UAVs in the IoRT environment. The study in [41] proposes integrating IoT, 5G, UAVs, and satellites to address IoT deployment challenges and overcome terrestrial infrastructure limitations, such as limited coverage and capacity. Nevertheless, while it provides a general architectural framework, it does not adequately address the challenges of integrating UAVs, satellites, and terrestrial 5G infrastructure, nor does it address the energy management complexities of NTNs.

The authors of the article [42] explore the potential of Artificial Intelligence (AI) techniques, including Machine Learning (ML) and Deep Learning (DL), for enabling ultra-reliable and low-latency communications (URLLC) and ubiquitous interconnectivity in the NTN-based Industrial Internet of Things (IIoT). However, the study primarily focuses on AI-based solutions and does not fully address the diverse range of challenges presented by IoRT use cases. In [43], the authors propose a simplified approach to analyzing Hybrid Satellite–Terrestrial Networks (HSTNs) by introducing three fundamental cooperative models, providing a survey of the state-of-the-art technologies for each model, and outlining prospective research directions. Nevertheless, their research specifically targets a subset of possible IoRT architectures.

The merits and drawbacks of current satellite-based IoT solutions for remote regions are highlighted in [44], which examines a variety of architectures and technical approaches. This study does not adequately address the diverse energy efficiency challenges associated with NTNs, and while it focuses on satellite-based IoT architectures, it does not consider the potential use of UAVs in the context of IoRT. Kua et al. in [45] provide a comprehensive overview of the potential benefits and challenges of using IoT and space-based technologies for future space exploration missions. While detailed, this study is focused on IoT for space and, therefore, does not consider all the range of IoRT scenarios and the respective challenges they pose. The study in [24] explores the need for hybrid satellite–terrestrial maritime networks, highlighting technologies that enhance efficiency, expand coverage, and support specialized maritime services. This study primarily focuses on the IoT implementations for maritime environments and thus does not comprehensively address the diverse landscape of IoRT scenarios.

The study [46] explores the integration of UAVs into Wireless Sensor Networks (WSNs). The authors discuss the performance and capabilities of UAVs as communication nodes, examine architectural aspects and emerging technologies within UAV-enabled WSNs (U-WSNs), and shed light on crucial factors that influence the design of U-WSNs. The study’s scope is limited to UAVs, and thus, it does not investigate satellite systems within the framework of IoRT. In [47], the authors examine the growing potential of NTNs in 5G and beyond networks, particularly when combined with terrestrial networks. The survey covers various aspects, including services, architectures, technological enablers, and challenges associated with NTN integration. Although NTNs are explored in this study, they are not evaluated in the IoRT context; hence, the associated challenges are not investigated.

Furthermore, while the study in [48] provides a comprehensive overview of the rapidly evolving landscape of wireless technologies, its focus on the broader IoT domain limits its ability to delve into the specific details and challenges of IoRT connectivity. The paper’s coverage of key performance indicators such as scalability, energy efficiency, reliability, and low latency is valuable, but its broad scope hinders a thorough examination of these factors within the unique context of IoRT applications. Additionally, the paper’s discussion of IoRT connectivity scenarios, particularly those involving satellite communication, has limitations due to its overarching focus on IoT as a whole.

Moreover, the study in [49] explores resource management for integrated space–air–ground–sea 6G networks incorporating UAVs and satellites, providing valuable insights. However, the study takes a general approach to these networks and does not specifically address the intricacies of IoRT environments. Moreover, it lacks adequate emphasis on energy-efficient strategies, particularly in utilizing Non-Orthogonal Multiple Access (NOMA) schemes and applying machine learning techniques such as Deep Reinforcement Learning (DRL).

Finally, the study in the work [50] offers a thorough analysis of challenges in satellite IoT networks, focusing on broad connectivity, extensive geographical coverage, mobility, and power consumption limitations. However, its focus remains exclusively on satellite NTNs, omitting any discussion of hybrid satellite-UAV and pure UAV connectivity approaches in IoRT environments. Furthermore, the study lacks a comprehensive analysis of energy-efficient approaches, especially through the utilization of machine learning techniques.

### Survey Scope

Therefore, as evident from the preceding discussion, a significant research void exists in the relevant literature, defining the domain that this current work aims to address: the realm of connectivity within the IoRT context, with a particular focus on the crucial aspects of energy efficiency and sustainability. Regarding the paper structure, Section 3 provides a detailed description of the characteristics of non-terrestrial networks, laying the foundation for understanding their significance in the context of IoRT. Moving forward, Section 4 identifies and clarifies the key challenges involved in integrating NTNs within IoRT systems. To address these challenges, Section 5, Section 6 and Section 7 present comprehensive surveys of IoRT systems based on Space-borne NTN Networks, Aerial NTN Networks, and Hybrid NTN Networks, respectively. Each survey investigates the unique objectives, characteristics, and challenges associated with these NTN implementations in IoRT. Section 8 examines future research trends and challenges in IoRT, while Section 9 focuses on the 3GPP standardization roadmap, emphasizing the importance of research and standardization efforts in both bridging remote connectivity gaps and fostering energy-efficient IoRT environments in the 6G landscape. Finally, Section 10 concludes the paper by summarizing our work and identifying future research directions.

## 3. Non-Terrestrial Networks

**Figure 1:**
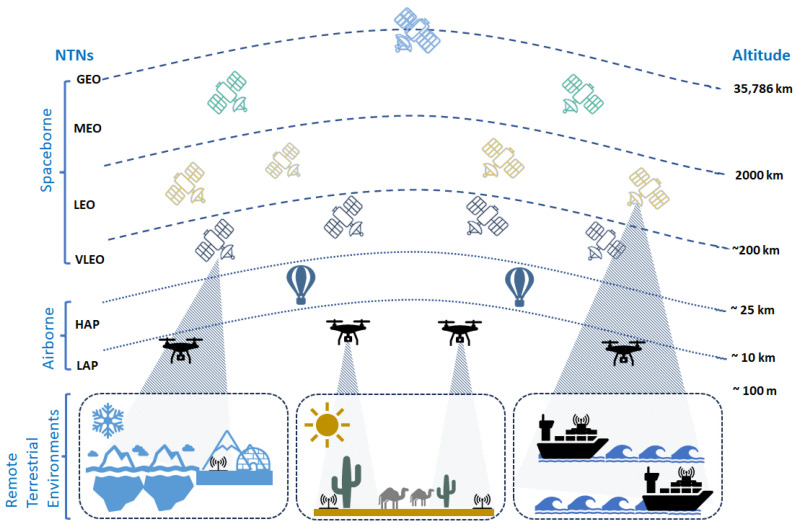
Non-terrestrial networks.

Current terrestrial networks face limitations in providing extensive wireless coverage to remote regions, adequate availability and reliability, and resilience to natural and man-made disasters. Non-terrestrial base stations, on the other hand, offer several advantages over their terrestrial counterparts. Their ability to be quickly deployed in emergency situations is crucial for disaster response, environmental monitoring, and search-and-rescue operations [51]. Additionally, their elevated positioning above the ground enhances radio link quality, reducing signal attenuation and interference from terrestrial obstacles. In essence as shown in Figure 1, the integration of terrestrial and non-terrestrial networks provides a 3D strategy for network coverage, paving the way for seamless and reliable services in remote areas, high-altitude locations, and deep-sea environments [52,53,54].

- *Space-Borne Networks Based on Satellites*
  Satellites operate in different orbits, each offering distinct advantages and limitations. The choice of orbit depends on the specific requirements of the IoT application. Geostationary Earth Orbit (GEO) satellites maintain a fixed position over the Earth, providing broad coverage but suffering from high latency and signal attenuation. LEO satellites, on the other hand, operate at lower altitudes (500–2000 km), minimizing latency and attenuation but requiring a larger constellation for global coverage [55,56]. Medium Earth Orbit (MEO) satellites offer a balance between coverage and latency, with a smaller constellation than LEO but a higher latency. Very Low Earth Orbit (VLEO) satellites, operating at altitudes below 200 km, promise ultra-low latency and attenuation but require advanced propulsion systems due to increased atmospheric drag and suborbital launch capabilities [57]. LEO and VLEO orbits are commonly utilized for deploying Cubesats, which are small, standardized satellites with a cube-shaped form factor [58,59]. CubeSats are often employed for educational and scientific purposes due to their compact size and lower launch costs. Nanosats, a broader category of small satellites that includes CubeSats but extends to slightly larger satellites, are suitable for a wider range of applications [60].
- *Airborne Networks Based on Unmanned Aerial Vehicles*
  Unmanned Aerial Vehicles (UAVs) offer a promising solution for expanding wireless coverage in challenging environments and beyond terrestrial infrastructure. They can serve as mobile base stations or relay nodes, bridging connectivity gaps and extending coverage to remote or isolated areas. This flexibility addresses the limitations of traditional IoT communication technologies, which often struggle in harsh or geographically constrained conditions [61,62]. UAVs can seamlessly integrate into existing terrestrial networks, effectively utilizing existing base stations without the need for additional infrastructure deployment. The altitude at which UAVs operate determines their role in the network: low-altitude platforms (LAPs) operate at altitudes of up to approximately 17 km, providing localized coverage and relaying data between terrestrial networks and remote areas. High-altitude platforms (HAPs), operating at altitudes of up to 25 km, offer broader coverage and can serve as central nodes for large-scale wireless systems [46].
- *Hybrid Non-Terrestrial Networks*
  Hybrid Non-Terrestrial Networks (Hybrid NTNs) refer to advanced communication infrastructures that seamlessly integrate multiple non-terrestrial technologies to create a unified and resilient network. This integration typically involves combining satellite communication systems with UAVs, either LAPs or HAPs [63]. The goal is to leverage the unique strengths of each component to enhance network performance, coverage, and flexibility. Satellites form the backbone of the network, providing global coverage and enabling long-range data transmission. UAVs and HAPs supplement this backbone infrastructure with dynamic and mobile capabilities, allowing for targeted coverage, rapid deployment, and persistent communication in specific areas. This synergistic combination of global reach and agility ensures the network’s ability to adapt to changing communication requirements and effectively serve remote and dynamic environments [64,65].

In conclusion, the utilization of non-terrestrial networks presents significant potential for expanding communication capabilities, enhancing coverage, facilitating the distribution of computing resources, reducing data processing delays, and establishing local IoT networks that can adapt to diverse Quality of Service (QoS) demands [66]. These platforms are characterized by their rapid and flexible development, which further enhances their ability to adapt to remote environments. In this context, the implementation of NTNs can facilitate the realization of different scenarios and services related to the IoRT ecosystem [47]. This involves providing support for demanding applications, including remote infrastructure control, monitoring, data collection, and connectivity, specifically in environments characterized by their remoteness, such as the Arctic, maritime regions, and rural areas.

## 4. Key Challenges in the Integration of NTNs in IoRT

**Figure 2:**
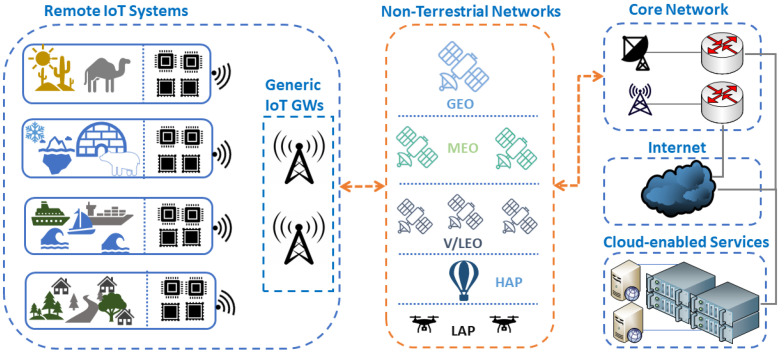
Generic IoRT System Model.

The Internet of Remote Things represents a groundbreaking advancement in IoT technology, extending its reach to connect remote and resource-constrained devices beyond the limitations of traditional terrestrial networks. Leveraging NTNs such as satellites, UAVs, and hybrid combinations of these platforms enables the collection, transmission, and processing of data from remote locations, unlocking a vast array of applications in diverse fields. Figure 2 illustrates a generic IoT system model for interfacing remote IoT systems to backbone communication infrastructure.

**Figure 3:**
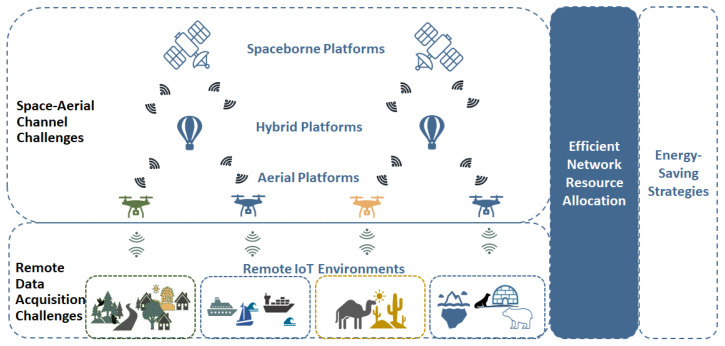
Key challenges in NTN-based IoRT systems.

Despite significant advancements in Non-Terrestrial Networks, integrating them into the Internet of Remote Things presents notable challenges, as shown in Figure 3. The utilization of space and aerial channels in NTNs, poses unique challenges in implementing underlying communication across diverse settings such as oceans, maritime zones, the Arctic, and rural areas. While enhancing the quality of space-aerial channels holds promise for extensive coverage and increased throughput, it confronts challenges related to signal propagation, atmospheric conditions, and latency. Hence, developing innovative approaches is essential to ensuring uninterrupted and reliable communication [67].

Furthermore, data collection in geographically isolated areas presents a distinctive set of challenges in ensuring timely and reliable data transmission and analysis. The interplay between spatial and temporal dynamics in these remote ecosystems can be intricate [68]. For instance, in a remote forest, the distribution of plant species may be influenced by geographic factors such as elevation and soil type as well as temporal factors like seasonal changes in temperature and precipitation. Due to the complexity of remote ecosystems, traditional methods of data collection, transmission, and analysis are often inadequate. Novel approaches are required, and new technologies and protocols are being developed to address these challenges [69].

Moreover, efficient resource allocation is always a paramount challenge, driving the development of advanced techniques to optimize resource distribution on satellite, UAV, or hybrid platforms [70]. This necessitates addressing obstacles related to scalability, prioritization, and adaptability in resource allocation. Energy efficiency especially poses a critical challenge, demanding sustainable and efficient energy management strategies for NTNs operating in remote and challenging environments [71]. Tackling these issues is crucial for the seamless integration of NTNs into the IoRT ecosystem, enabling efficient communication across a wide range of applications in remote and challenging conditions [72]. In our classification of papers on non-terrestrial networks for the IoRT, we identified seven important objectives. While specific objectives might overlap and impact one another, we centered on each work’s main research focus.

1. *Energy Efficiency*: NTNs play a crucial role in extending the lifespan and reducing the operational costs of IoT devices, especially in remote or resource-constrained environments. Research efforts aim to optimize NTNs to minimize energy consumption while maintaining network performance.
2. *Cost-Efficient Deployment/Operation*: The deployment and operation of NTNs, particularly in large-scale IoT deployments, must be cost-effective. Researchers explore strategies to reduce infrastructure costs, optimize network utilization, and minimize maintenance expenses.
3. *Enhanced Coverage and Availability*: NTNs provide an opportunity to extend network coverage beyond the limitations of terrestrial networks, ensuring reliable connectivity in challenging terrains and remote areas. Research focuses on improving NTN coverage, overcoming obstacles, and maintaining connectivity in harsh conditions.
4. *Enhanced Throughput/Transmission Rate*: As IoT data volumes continue to grow, NTNs need to handle high-speed data transmission to meet the demands of applications. Researchers investigate techniques to increase NTN throughput and support efficient data transfer.
5. *Enhanced Data Transmission Reliability*: Reliable data transmission is paramount, especially for mission-critical IoT applications. NTN research addresses factors such as interference, fading, and signal attenuation to ensure data integrity and minimize packet loss.
6. *Enhanced and Timely Data Acquisition*: IoT devices generate vast amounts of data at frequent intervals. Timely data acquisition is crucial for proper processing and analysis. NTN research explores methods to optimize data collection, reduce delays, and ensure timely data delivery, often leveraging advanced Medium Access Control (MAC) protocols to optimize channel access.
7. *Enhancement of Edge Computing Capabilities*: Edge computing brings processing power closer to IoT devices, reducing latency and enabling real-time decision-making.

By addressing these objectives, NTNs for IoRT are poised to revolutionize the way data are collected, processed, and utilized, enabling a wide range of applications in remote and isolated environments.

## 5. IoRT Systems Based on Space-Borne NTN Networks

This category of satellite-based IoRT systems is the most prevalent, as it represents the most fundamental approach to establishing remote connectivity. This can be achieved through either the direct connection of remote devices to the satellite or through the establishment of a local network that utilizes a central communication gateway to interface with the satellite. Therefore, in this category, we identified 54 related research works that are classified according to their main research objective into the following subsections.

### 5.1. Enhancement of Energy Efficiency

In [73], the contribution of satellites to cost-effective solutions for a large number of users and devices in the emerging 5G cellular system is highlighted. Given the broadcast nature of satellite communications, access to remote areas, and support for multiple devices, satellites are positioned to play a significant role in the development of the Internet of Things industry. The purpose of this paper is to investigate Network Coding (NC) techniques within a hybrid satellite/terrestrial network, specifically to improve the reliability of multicast services. The authors propose an energy-optimization method involving adjustments in data symbol repetition and coded packet numbers based on the group’s needs and the prevailing satellite link conditions, with a focus on software updates, control messages, and the efficient delivery of content to multiple devices.

The authors of [74] propose a novel random access preamble design and detection method to address the energy-intensive nature of random access for IoT devices in LEO satellite-based NTNs. Their approach eliminates the need for additional signaling and simplifies the detection process. They also present and employ a metric that effectively mitigates the impact of carrier frequency offset and reduces noise-induced timing estimation errors. The proposed metric’s statistical analysis reveals that increasing correlation length improves the output Signal-to-Noise Ratio (SNR) power ratio, and the first-path detection threshold is independent of noise statistics. Simulation results in various LEO scenarios demonstrate the proposed method’s robustness in achieving considerable improvements, particularly in terms of energy, when compared to existing random-access methods.

Power-efficient scheduling for NTNs to support IoT applications is the focus of the study in [75]. In order to overcome the challenges posed by the typical duty-cycled nature of IoT applications and the limited satellite access time, this article proposes a method for synchronizing sleep and wake-up periods with satellite availability. By reducing the frequency of satellite orbital information updates mandated by Simplified General Perturbation 4 (SGP4), this approach has the potential to substantially enhance the battery life of IoT-NTN devices. Data from a real communication link between a terrestrial device and a LEO CubeSat is utilized to validate the proposed method.

The authors in [76] propose utilizing collaborative LEO satellites to enhance IoRT systems. This approach involves combining uplink signals from multiple LEO satellites, substantially improving SNR, and reducing power requirements for energy-constrained terminals. The paper outlines a comprehensive system design, encompassing frequency planning, waveform selection, collaboration strategies, and terminal design. A coherent combining scheme is introduced to address signal reception impairments, and a modified SUMPLE algorithm is presented for phase difference compensation and estimation. Simulation results validate the effectiveness of the proposed algorithms, particularly in low SNR conditions.

A novel MEC framework for terrestrial-satellite IoT utilizing LEO satellites and terrestrial-satellite terminals is proposed to enable computation outsourcing from IoT Mobile Devices (IMDs) [77]. This framework employs a two-tiered subproblem approach to minimize the weighted-sum energy consumption of IMDs. The lower layer utilizes sequential fractional programming to minimize space segment latency, while the upper layer employs a convex structure and Lagrangian dual decomposition. Based on solutions to these subproblems, an Energy-efficient Computation Outsourcing and Resource Allocation algorithm (E-CORA) is introduced. Simulation results demonstrate that E-CORA effectively reduces the energy consumption of IMDs by optimizing bit offloading, outperforming both full bit offloading and local computing.

Resource allocation remains a critical challenge in satellite IoT deployments, particularly in remote areas. However, existing strategies often fail to adequately address the unique characteristics of low-earth-orbit satellite systems, such as mobility and energy constraints. In [78], the authors propose DeepCA, a Deep Reinforcement Learning-based (DRL) method for energy-efficient channel allocation. DeepCA effectively tackles the challenge of limited channel resources in ground-based nodes, leading to a significant reduction in energy consumption compared to conventional approaches.

The growing demand for IoT connectivity necessitates enhanced spectrum and energy efficiency in satellite-based IoT systems. The work in [79] proposes a novel power control algorithm for IoT terminals in satellite-based networks, leveraging terrestrial base stations for data acquisition and resource management. By employing the Poisson Point Process (PPP) theory, the optimization problem is formulated considering the distribution of IoT devices within the network. The optimal power control scheme is determined by optimizing user distribution and Signal-to-Interference-plus-Noise Ratio (SINR) demand. To address the complexity of the PPP-based objective function, the pattern search procedure is employed. Numerical evaluations of user rates and energy efficiency demonstrate the effectiveness of the proposed power control algorithm.

Furthermore, the study in [80] introduces multi-connectivity to multi-orbit NTNs, allowing user terminals to connect to numerous satellites in order to increase peak throughput. The researchers propose a terminal-aware multi-connectivity scheduling algorithm that optimizes uplink data rates and minimizes energy consumption at the user terminal based on radio availability and propagation data. Operating within a differentiated multi-layer NTN resource scheduling architecture, the efficacy of the algorithm is compared to that of other scheduling methods, evaluating the uplink data rate and energy efficiency. In addition, they present an architectural design for implementable schedulers in multi-orbital satellite networks that can accommodate a variety of terminal classes.

In [81], the authors explore the challenges of enabling massive access in a Beyond-Fifth-Generation (B5G) satellite IoT network using a multibeam architecture. They focus on the issue of channel phase uncertainty caused by the transmission of channel-state information from devices to satellites through gateways. To overcome this challenge, they propose a novel NOMA scheme that effectively supports the wide-scale deployment of IoT devices. Considering the energy limitations of LEO satellites, they introduce two robust beamforming algorithms to minimize total power consumption for non-critical and critical IoT applications while taking channel phase uncertainty into account. Theoretical analysis and simulations demonstrate the effectiveness and robustness of these algorithms in enabling massive access in the satellite IoT.

The authors of [82] propose a DRL algorithm to manage channel allocation and power control in the satellite IoT uplink scenario. The algorithm aims to optimize resource utilization and service quality. Simulations demonstrate that the approach successfully achieves an efficient balance between power efficiency and service quality. Under typical scenarios for satellite IoT, the proposed scheme greatly improves power efficiency in comparison to current methods, with minimal training overhead.

In conclusion, to enhance the energy efficiency of IoRT systems based on space-borne NTN networks, various techniques have been proposed, ranging from utilizing the repetition factor of data symbols to DRL-based methods for dynamic and energy-efficient channel allocation. Table 3 presents a concise summary of the information presented in this section.

### 5.2. Cost-Efficient Deployment/Operation

The study in [83] tackles the challenge of residual Doppler shifts in LEO satellite communications for NB-IoT over LEO NTN systems. The proposed technique combines demodulation reference signal symbols and reduced satellite beam coverage to compensate for residual Doppler shifts. Link-level simulations and link budget analysis for actual three-dimensional orbits demonstrate that the proposed method significantly reduces the number of required satellites while maintaining continuous communication, positively impacting both the total service time and the most persistent service time.

Furthermore, as described in [84], the Direct-to-Satellite Internet of Things (DtS-IoT) concept employs satellite constellations as gateways in orbit for a global IoT network. The research emphasizes the suitability of particular Long-Range (LoRa) network protocol configurations for achieving this objective. As a consequence, the idea of sparse satellite constellations emerges, significantly reducing in-orbit infrastructure while only marginally increasing latency. An algorithmic approach incorporating specific heuristics is proposed to generate quasi-optimal topologies for sparse IoT constellations. The results indicate that LoRa-enabled DtS-IoT services can be enabled on a global scale with substantially fewer satellites than traditional constellations require.

CubeSats offer a novel method for achieving global connectivity by providing cost-efficient and wide geographic coverage. In order to overcome existing constraints and optimize network management, the authors of [85] propose the adoption of Software-Defined Networking (SDN) and Network Function Virtualization (NFV), which would allow for precise control over hardware and network resources. The study provides a thorough and detailed description of the design and components of the system, addressing the distinct challenges presented by the space environment. An initial performance test, which focuses on important measurements such as latency and throughput, highlights the significant potential of this technology in the IoRT domain.

The implementation of a Software-Defined Radio (SDR)-based satellite gateway is proposed in [86] as a solution to the challenges that arise from the diverse communication standards associated with IoRT applications. Utilizing SDR’s capacity to decrease hardware expenses and improve adaptability, this approach maximizes efficiency. The proposed architecture was developed utilizing a standalone SDR platform and Commercial Off-The-Shelf (COTS) modules and is able to support the main IoT telecommunication standards.

Satellite communication systems, particularly those utilizing LEO satellite constellations, offer a cost-effective and scalable solution for global IoT applications, enabling ubiquitous connectivity across vast geographical regions with minimal infrastructure deployment. The study in [87] delves into the optimization of uplink transmission scheduling within LEO satellite networks to address the challenge of limited bandwidth resources. The proposed algorithm, a novel hybrid of Simulated Annealing and Monte Carlo (SA-MC) techniques, dynamically adapts to varying data traffic demands and network conditions, achieving significant cost reductions and rapid convergence.

CubeSat platform satellites are introduced in [88] as an attempt to provide low-cost, extensive coverage for IoT/Machine-to-Machine (M2M) services in remote areas. In particular, they investigate the challenges of the earth–space communication link in the context of IoT/M2M. Their system model consists of Machine-Type Devices (MTDs), LEO satellites, and ground stations (i.e., gateways). The MTDs serve as data sources since they provide uplink signals to satellites, which then transmit the data to the first gateway. However, due to the highly variable uplink channel load and the limited communication cycle of MTD, an ineffective channel reserve system results. Therefore, they propose an energy-efficient multi-access technique for M2M communication in IoRT systems and develop an energy-efficient random access protocol for delay-tolerant applications.

Table 4 summarizes recent research on cost-effective deployment and operation of IoRT systems based on space-borne NTN networks. The studies proposed various solutions, including compensating for residual Doppler shifts, investigating LoRa protocol variations, using CubeSat SDN NFV-based architectures, employing SDR, and developing efficient uplink scheduling algorithms. The proposed solutions aim to minimize satellite hardware requirements, reduce gateway costs, and optimize the utilization of transmission resources, leading to more cost-efficient IoRT systems.

### 5.3. Enhanced Coverage and Availability

To address the growing demand for global connectivity in the IoT realm, ref. [89] explores the potential of beam-hopping-based satellite systems. Tailored for IoT applications, these systems offer efficient coverage expansion and adaptability to dynamic traffic patterns, making them well-suited for massive machine-type communication scenarios. By incorporating adaptations for beam scheduling, initial access, and data transmission, the proposed scheme effectively addresses the unique requirements of IoT communication. Numerical analyses reveal the system’s superior performance in terms of battery life, link budget, and system capacity, paving the way for optimized resource allocation across diverse IoT use cases.

In an effort to address the issue of unreliable internet connectivity in remote or underserved regions, a solar-powered satellite internet access point is proposed in [90]. By utilizing solar energy, this innovative Access Point (AP) ensures a reliable internet connection, especially in areas where the electricity supply is irregular. It ensures that sensors, IoT devices, and other technological equipment have continuous access to the internet. Moreover, it prioritizes efficiency in terms of costs and ease of maintenance through a meticulous selection of components. Its ability to maintain internet connectivity for a duration of four days without solar power is particularly notable.

In [91], the researchers propose a novel approach for evaluating poor connectivity due to latitude shifts while maximizing the number of IoT devices that can be synchronously accessible despite coverage variations. To achieve this, they developed a novel coverage level measurement method that is used to evaluate the average coverage level within a return circle of an LEO satellite constellation at varying distances. Furthermore, they used a spot beam communication technique with Time Division Multiple Access (TDMA) as a discrete-time mechanism to determine the maximum number of devices within a unit area that can be accessed during a time frame. Finally, they studied the relationship between device density, maximum delay tolerance, and satellite constellation coverage degree.

Integrating LEO satellite constellations with terrestrial networks to support IoRT services requires careful placement of gateways to maximize network coverage, minimize access distance, and maximize revenue. The authors in [92] propose a novel gateway placement algorithm that considers both communication and IoRT service data demands, as well as channel conditions and service demand distribution, and ensures efficient network resource utilization while safeguarding local information confidentiality. Simulation results demonstrate the algorithm’s improvement in resource utilization and coverage performance. Additionally, the findings highlight the significance of service demand distribution in gateway placement decisions.

A comprehensive modeling framework for IoT-over-satellite access systems is introduced in [93]. This framework considers a multitude of factors, including satellite orbit, uplink interference from terrestrial IoT devices, atmospheric conditions, gas absorption, and the probability of line-of-sight. The efficacy of the system is evaluated based on the uplink SINR and satellite link time availability during a pass. Focusing on satellites in low-earth orbit, the study employs actual orbital parameters ranging from 300 to 800 km. Additionally, a numerical model for optimizing antenna beamwidth is proposed to maximize link availability while minimizing terrestrial interference and enhancing the IoT signal.

The authors of [94] introduce a Ka-band multibeam satellite industrial IoT framework to overcome the limitations of conventional ground-based IIoT, particularly its limited coverage range. By employing NOMA within each beam, they aim to optimize transmission rates for enhanced QoS. Resource allocation is maximized by optimizing beam power to align theoretical transmission rates with service demands. Moreover, the study proposes a satellite-ground integrated IIoT solution, utilizing terrestrial cellular networks to extend satellite coverage in obstructed areas while incorporating transmission cost reduction strategies. Simulation results validate the benefits of NOMA in satellite IIoT.

Table 5 summarizes the research on enhancing the coverage and availability of IoRT systems based on space-based NTN networks. These advancements include the development of beam-hopping-based satellite systems for IoT, solar-powered satellite access points, algorithms for optimal gateway placement, modeling frameworks for maximizing link availability, and novel multibeam satellite architectures. These innovations have the potential to significantly expand the reach of IoRT applications to remote and underserved areas, paving the way for a more interconnected and globally accessible IoT ecosystem.

### 5.4. Enhanced Throughput/Transmission Rate

Although the implementation of satellite connectivity for IoRT devices shows potential, it also poses difficulties in terms of spectrum management for 5G satellite networks. In response to these challenges, ref. [95] presents an innovative, dynamic spectrum management scheme that is tailored to this particular context. The effectiveness of the proposed scheme in spectrum management for machine-to-machine and human-to-human communications in 5G satellite networks is demonstrated by simulation results on throughput, delay, spectral efficiency, and fairness index.

NB-IoT, a promising technology for Low-Power Wide-Area Networks (LPWANs), enables widespread deployment of IoT services. However, in remote areas, terrestrial coverage gaps necessitate the integration of LEO satellites as a supplementary network layer. However, the inherent differential Doppler shift associated with satellite communication can degrade system performance. Authors [96] delve into the NB-IoT over LEO satellite architecture, where the satellite provides NB-IoT connectivity to fixed NB-IoT terminal equipment located beyond the reach of terrestrial infrastructure. To address the significant differential Doppler among terrestrial user channels, the authors propose a resource allocation scheme that effectively mitigates excessive Doppler values. Specifically, they propose segmenting the coverage area into smaller sub-regions, ensuring that the differential Doppler within each region remains within permissible limits.

A phased-array antenna framework for IoT-over-satellite applications leveraging nano-satellites is presented in [97] to facilitate global IoT connectivity in remote areas. The proposed approach enables precise beamforming toward targeted IoT devices, boosting received signals while minimizing interference from other terrestrial devices. By efficiently sharing radio spectrum resources, this approach enhances capacity. A practical implementation of this framework involves fabricating an X-band phased array antenna, which has been validated through gain pattern and return loss testing, confirming its feasibility.

In the realm of the satellite-based Internet of Things, efficient access for numerous sensors and short-burst transmissions remains a challenge. To address this issue, ref. [98] proposes a time-slot random access protocol employing Walsh codes specifically tailored for sink node scenarios. A load estimation-based, dynamic Walsh code selection mechanism is introduced to optimize system throughput. Simulation results demonstrate the protocol’s effectiveness in enhancing system throughput under high load conditions while effectively managing resource utilization during periods of medium and low demand.

The research in [99] explores a Non-Orthogonal Multiple Access-based multi-user Beamforming (NOMA-BF) scheme for improving spectral efficiency in satellite-based IoT. The proposed scheme has been developed for scenarios in which primary users are prioritized with timely data transmissions and guaranteed data rates, while secondary users are opportunistically served. To reduce inter-beam interference, improve effective channel gains, and optimize inter-beam power allocation, the scheme employs a multi-layered beamforming strategy. Numerical results demonstrate that the proposed scheme enhances spectral efficiency while meeting user data rate requirements.

The research work in [100] emphasizes the potential of the upcoming 6G standard to provide seamless IoT network coverage by 2030. Underscoring the importance of satellite-based communications in meeting IoT service requirements in the 6G era, this study analyzes in depth the multiple access technologies required for effective satellite-based IoT deployment. It includes information-theoretic considerations and key technologies such as NOMA and Random Access (RA). This analysis evaluates how the satellite transmission environment impacts these access technologies. Furthermore, it aims to improve system throughput and robustness under varying traffic conditions by proposing a novel Non-Orthogonal Massive Grant-Free Access (NoMaGFA) scheme that combines the benefits of RA and NOMA for asynchronous transmissions in satellite-based IoT while maintaining low signaling overhead.

In response to the growing demand for spectrum-efficient satellite-based IoT systems, ref. [101] introduces a cognitive satellite-terrestrial framework that leverages multibeam satellite capabilities with full frequency reuse. The proposed scheme maximizes the achievable rate while ensuring outage probability and power consumption constraints. To address the complex optimization problem, a two-level iterative algorithm is developed that incorporates Bernstein-type and large deviation inequality-aided methods for probabilistic constraint handling. Numerical simulations illustrate that the proposed scheme is efficient as well as sensitive to key parameters.

The study in [102] identified NB-IoT as a key technology for supporting massive machine-type communication (mMTC) scenarios within 5G networks. LEO satellites offer the potential to enhance the link budget by reducing propagation signal loss, which is crucial for low-complexity, low-power, and cost-effective IoT devices. However, LEO orbits introduce higher Doppler effects, which can negatively impact the performance of IoT communication. To mitigate these effects, the study proposes an uplink scheduling technique for a mMTC-NB-IoT system based on LEO satellites. Numerical simulations are employed to evaluate the performance of the proposed technique and highlight the limitations imposed by the satellite channel.

Table 6 presents a concise summary of the information presented in this section. Various techniques, such as efficient spectrum management, beamforming, and NOMA, are employed to achieve significant improvements in data transmission rates. These techniques address the challenges posed by large-scale IoT deployments, paving the way for future IoRT systems that can support massive connectivity.

### 5.5. Enhanced Data Transmission Reliability

Researchers in [103] examine the importance of satellite Machine Type Communication (MTC) in the maritime IoT and highlight the challenges hindering its implementation. Their focus is on investigating potential interference issues between a satellite MTC system for maritime IoT and co-frequency terrestrial communication systems already in operation. The main obstacle stems from the lack of established regulatory criteria and methods to safeguard terrestrial systems against potential interference from LEO satellite systems or similar technologies. This absence makes it challenging to assess the impact on existing land systems. The researchers contribute significantly by mathematically deriving an electromagnetic Power Flux Density (PFD) mask. This mask helps evaluate and limit satellite space station emissions within the existing regulatory boundaries designed for mitigating interference among terrestrial communication systems in the same frequency band. The principle guiding this approach is ensuring that the interference experienced by a land system due to a space station is no more severe than that from a co-frequency land system permitted by regulations.

Addressing the challenging link between IoT devices and GEO satellites, a novel modulation and signaling scheme based on Chirp-Spread Spectrum (CSS) is proposed in [104]. This scheme, termed Unipolar-coded CSS (UCSS), enables reliable transmission at ultra-low bit rates and supports true random multiple access for a large number of devices, even at high carrier frequencies such as C-band to Ka-band. This breakthrough paves the way for ubiquitous connectivity and mMTC via satellite, even in remote and underserved areas. Furthermore, in [105], the same authors further delve into the challenges and opportunities associated with direct access of IoT devices to GEO satellites. They highlight the potential of UCSS to revolutionize satellite-based IoT communication, enabling reliable and efficient connectivity for a wide range of IoT applications. The successful demonstration of UCSS with a Ku-band testbed validates its feasibility and opens up exciting possibilities for ultra-narrow band mMTC via satellite.

The study in [106] highlights the transformative potential of satellites to significantly enhance the performance of IoRT networks, enabling seamless connectivity and robust data transmission across remote areas. By leveraging a Reinforcement Learning (RL) framework, the authors effectively address the challenge of optimizing resource allocation and IoRT data scheduling in dynamic and unpredictable environments. To overcome the limitations of traditional RL algorithms, they propose customized feature functions tailored to the unique characteristics of satellite-based IoRT networks and employ function approximation techniques to enhance the accuracy of resource allocation decisions. Their proposed state-action-reward-state-action-based RL strategy proves highly effective in simulations. The utilization of a linear combination of proposed features specifically designed for decision-making and the prevention of battery overflow results in effective energy management and reduced energy consumption.

Excessive propagation delay is also a significant challenge in satellite-based IoT (S-IoT). In this particular context, conventional Hybrid Automatic Repeat request (HARQ) strategies are frequently unsuccessful as a result of limited feedback efficiency. To tackle this challenge, a novel Network Code HARQ (NC-HARQ) transmission protocol is introduced in [107]. Enabling efficient multi-hop communication is its primary objective, particularly in S-IoT environments where feedback mechanisms are inadequate or absent. In order to evaluate the performance of the NC-HARQ protocol in terms of Age of Information (AoI), a four-state Markov model is utilized. This model facilitates the derivation of closed-form expressions for the mean AoI in the context of end-to-end two-hop transmission. The results of the simulation illustrate that the NC-HARQ protocol is more effective than several modern HARQ schemes, as indicated by the significantly decreased average AoI levels.

In [108], the authors highlight the very limited choice of available technologies and the challenges of developing data collection and control systems in remote areas. They conduct a critical review and analysis of various protocols and technologies used for IoT data transmission, and they propose a hybrid IoT-satellite network to address these limitations. This network intends to gather data employing a terrestrial LoRa LPWAN, with backhaul connectivity provided via the Iridium satellite system. Different data presentation formats for low-speed satellite channels were evaluated through simulations. Furthermore, the authors introduce GDEP (Gateway Data Encoding and Packaging), a data encoding and packaging technique that is able to reduce the number of Short Burst Data (SBD) containers, improving network efficiency.

In order to improve the efficiency of accessing and utilizing resources in a massive machine-type communication scenario, the authors of [109] suggest a collision avoidance technique as the first step in random access for LEO satellite Internet of Things. The proposed method allows for quick identification of collisions and accurate estimation of load while also being resistant to non-orthogonal interference. An analysis is performed on the probability of detecting the preamble, the probability of detecting collisions, and the accuracy of load monitoring. This analysis results in the development of an optimal set of probabilities for selecting preambles, which maximizes the precision of load monitoring.

To address the issue of data staleness caused by the slow sensor transmission rate and the high bit error rate of the satellite-to-ground link, ref. [110] introduces an age-optimal hybrid temporal-spatial generalized deduplication and automatic repeat request (HARQ-GD) protocol designed for high-sampling data collection in the satellite-integrated Internet of Things. By integrating temporal and spatial data correlations, encoding and decoding algorithms, and packet format optimization, the HARQ-GD protocol successfully mitigates data staleness. As a result, transmission times are reduced, and the compression rate is enhanced. The simulation results indicate that the HARQ-GD protocol reduces the Age of Information (AoI), a metric that quantifies the freshness of data, more effectively than conventional Generalized Deduplication (GD) and Hybrid Automatic Repeat Request With Chase Combining (HARQ-CC) schemes.

LEO satellite networks have emerged as a promising solution for ubiquitous IoT connectivity. A Grant-Free Random Access (GF-RA) mechanism is proposed in [111] to address the dynamic nature of IoT traffic and intermittent transmissions from randomly activated devices. This GF-RA system employs a modified Bernoulli-Rician Message Passing (BR-MP) technique with Expectation-Maximization (EM) for User Activity Detection (UAD) and Channel Estimation (CE). Extensive simulation results of the proposed BR-MP-EM algorithm validate its accuracy and show its resilience to channel impairments.

In [112], researchers propose an integrated satellite-terrestrial IoT network to extend coverage to areas not serviced by terrestrial cellular networks. The system employs Power Domain Non-Orthogonal Multiple Access for both LEO satellites and terrestrial base stations. However, interference arises between LEOs and base stations due to limited feedback line capacity. To mitigate this issue, researchers aim to minimize the overall network’s transmission power while ensuring acceptable IoT device communication rates. To tackle this challenge, an Iterative Penalty Function (IPF)-based scheme is proposed for designing robust beamforming for satellites and base stations. Simulation results demonstrate the effectiveness of the proposed scheme in satellite-terrestrial IoT scenarios.

Table 7 summarizes the proposed schemes to enhance the reliability of IoT data transmission. The mitigation of interference, the development of novel modulation and signaling schemes, the use of RL-based resource allocation methods, HARQ feedback enhancement, the design of novel data encoding schemes and collision detection techniques, and robust beamforming schemes for NOMA-based networks are among the highlighted approaches.

### 5.6. Enhanced and Timely Data Acquisition

The study in [113] explores the use of CubeSats as low-cost satellite network building blocks for the Internet of Space Things. To enable seamless functionality for a variety of use cases and stakeholders, an automated network slicing methodology is proposed for space-ground integrated networks. Designed for extremely dense CubeSat networks, the system focuses on effective route computation and resource allocation while ensuring Service Level Agreement (SLA) adherence. Notably, it employs an SLA-based methodology, eliminating the need for prior knowledge of slice resource requirements. Furthermore, a case-driven evaluation scenario was employed to evaluate the adaptability and effectiveness of the proposed framework.

The study in [38] addresses the challenge of establishing IoT connectivity in remote areas with restricted communication infrastructure, such as the Arctic. The research presents an alternative solution involving a swarm of small, freely drifting satellites and industry-standard protocols. The authors validate the feasibility of this approach by simulating networking protocols and link characteristics across different satellite orbits and ground nodes. The findings indicate that small-satellite swarms can significantly minimize communication overhead and end-to-end request latency, enabling IoT deployment even in the most remote settings, like the Arctic.

The researchers in [67] explored the use of a satellite-enabled IoRT network to collect data in remote areas with no internet connectivity. Specifically, the Xingyun satellite constellation was employed to gather environmental data from the Tibetan Plateau. The monitoring system consisted of terrestrial, ground-based terminals equipped with satellite transceivers and environmental sensors. Five such terminals were deployed in challenging regions to monitor air temperature, relative humidity, precipitation, snow depth, land surface temperature, tree stemflow rate, and photosynthetically active radiation. Field experiments evaluated the performance of the proposed system, revealing its efficiency.

The research in [114] focuses on the problem of maintaining fast data updates in satellite-integrated IoT networks for applications like animal tracking and environmental monitoring. To address this, the authors propose Spatially Temporally Correlative Mutual Information (STI), a new metric that takes into account correlations between the most recent update message and the current state of the data source. They optimize channel slot allocation using a Markov decision process framework by maximizing the averaged STI over a specified updating period. The simulation results show that the proposed strategy outperforms traditional scheduled access schemes such as slotted ALOHA and Threshold-ALOHA.

In [115], a packet scheduling algorithm designed specifically for IoRT devices operating in 5G satellite networks is proposed. The algorithm achieves its objectives through the implementation of a cross-layer design approach. The principal objective of this packet scheduler is to optimize system throughput while ensuring a reasonable degree of fairness for both delay-sensitive and delay-tolerant IoRT services. The simulation results indicate that the proposed packet scheduler demonstrates better performance in terms of throughput, spectral efficiency, and fairness index when compared to previously proposed schemes.

The expanding M2M/IoT traffic and its potential for satellite delivery are examined in [116]. The emphasis is on comparing two major M2M/IoT protocol stacks, Constrained Application Protocol (CoAP) and Message Queuing Telemetry Transport (MQTT), on a satellite Random Access (RA) channel using the DVB-RCS2 specification. The completion time metric is critical in determining which protocol stack is preferable. The study emphasizes the advantages of the Publish/Subscribe (PUB/SUB) paradigm, particularly in satellite-based architectures, for its effectiveness in rapidly delivering new data to subscribers. The authors then propose integrating CoAP with the observer pattern and proxying functionality to optimize the PUB/SUB paradigm. CoAP outperforms MQTT on RA satellite channels, demonstrating the adaptability of application-layer tuning options.

A QoE-aware satellite constellation design scheme is proposed in [117] to improve user experience in satellite IoT applications. The satellite IoT network is composed of LEO satellites and ground-based IoT devices. Quality of Experience (QoE) factors are defined to evaluate coverage performance, communication efficiency, regional demand capacity, and profitability. A Multi-Layer Tabu Search (MLTS) optimization algorithm is then used to determine optimal satellite orbits that maximize QoE. The simulation results show that the proposed constellation is effective in optimizing multiple QoE factors, resulting in a better user experience.

Within the context of IoRT, the authors of [118] address the challenge of interconnecting widely dispersed IoT nodes. They propose a cooperative mechanism that employs feedback on link conditions and adaptive coding knowledge to optimize resource allocation and improve video transmission quality and fairness among IoRT nodes. The proposed methodology was validated by implementing it on an emulation platform, demonstrating its efficiency and advantages over existing methods. This cooperative mechanism has the potential to improve the performance and reliability of IoRT networks, especially in disaster recovery scenarios where seamless video transmissions from remote locations are critical.

A study in [119] proposes a joint optimization strategy for maximizing long-term network utility in a NOMA Satellite IoT downlink system. The proposed approach employs two virtual queues to manage both data queuing and power consumption. Lyapunov optimization is employed in order to attain network stability and optimize resource allocation, taking into account the constraints of onboard communication resources. Moreover, the authors propose a solution utilizing Successive Interference Cancellation (SIC) decoding in conjunction with Particle Swarm Optimization (PSO) to determine the optimal resource allocation strategy. The simulation results show that the joint optimization allocation operates efficiently in terms of long-term network utility, average data rate, and queuing delay.

Table 8 summarizes studies that have explored the potential of integrating space-borne NTNs into IoT networks to enhance data acquisition and improve timeliness. These studies have proposed innovative approaches for network slicing, small-satellite swarms, and QoE-aware constellation designs. They have also evaluated the performance of different protocols and resource allocation mechanisms and demonstrated that space-borne NTNs are a promising solution for enabling delay-sensitive IoRT applications in remote and challenging environments.

### 5.7. Enhancement of Edge Computing Capabilities

Advancements in ultra-dense satellite communications, as explored in [120], could offer an alternative approach for expanding IoRT computational efficiency and resource utilization. IoRT devices can offload their processing tasks to satellite networks or relay them to terrestrial data centers via satellite communications. However, due to resource constraints, a single satellite cannot handle computation-intensive and complex operations. This paper proposes a collaborative task processing scheme that utilizes multiple satellites in a group to simultaneously process IoRT tasks, enhancing computational efficiency and resource utilization. To maximize completion delay reduction for all IoRT devices, the optimization problem is formulated as a Winner Determination Problem (WDP), and a low-complexity cooperative computation algorithm is presented as a solution.

Moreover, the growing importance of IoT in the information industry is emphasized in [121], highlighting the need to address network strain, particularly for remote IoT platforms. Edge computing is proposed as a solution for offloading processing closer to data sources. While satellite data transmission is crucial for remote IoT devices, conventional satellites lack versatility due to their specific application designs. To address this limitation, the authors propose transforming conventional satellites into adaptive space-edge computing nodes, enabling dynamic resource sharing and software management. Simulations demonstrate the efficiency of this approach compared to conventional satellite constellations, with the quality of service depending on the number of satellites, computational capacity, and task outsourcing strategies.

To address the challenge of managing outsourcing path selection and resource allocation for computation-intensive and delay-sensitive tasks in dynamic network environments, a novel Ka/Q-band satellite-terrestrial integrated network for IoT in remote locations is proposed in [122]. The problem is formulated as a Markov decision process, with the aim of maximizing offloaded tasks while minimizing LEO satellite power consumption and adhering to specific delay constraints. DRL techniques are applied in making optimal decisions by leveraging dynamic IoRT device queues and taking into account time-varying channel conditions and ground station computing capabilities. Simulation results demonstrate the effectiveness of the proposed scheme.

An integrated satellite-terrestrial network architecture is proposed in [123] to enhance delay-sensitive task outsourcing for IoRT applications. This architecture seamlessly integrates satellite and terrestrial networks, expanding communication resources, backhaul capacities, and coverage to address the challenges of IoRT deployment. A key focus of the proposed architecture is to optimize offloading link selection and bandwidth allocation for base stations and IoT users. To address the differentiated time granularities of various decision-making processes, a two-timescale stochastic optimization problem is formulated. The authors develop a Hybrid Proximal Policy Optimization (H-PPO)-based algorithm to efficiently address the subproblems involved in the optimization problem. Simulation results demonstrate the effectiveness of the proposed scheme, particularly in scenarios characterized by limited spectrum resources and heavy traffic volumes.

To enhance the performance of IoT applications in LEO satellite networks, the authors of [124] propose a hybrid offloading architecture that integrates satellite mobile edge computing, which enables IoT devices to directly access satellite-based computational resources, eliminating the need for distant cloud servers and minimizing transmission and computation energy consumption. This approach alleviates bandwidth constraints and minimizes transmission delays. Additionally, the authors present a multi-agent actor-critic RL algorithm to optimize outsourcing policy decisions while taking into account the limitations of satellite resources.

Table 9 summarizes the research efforts that have explored the use of space-borne NTN networks to enhance edge computing capabilities for IoRT applications. These efforts have demonstrated the potential of space-based NTNs to provide low-latency, high-bandwidth connectivity to remote IoRT devices, enabling the offloading of computation tasks to satellites for processing. This can significantly improve the performance and scalability of IoRT applications, particularly in resource-constrained environments.

## 6. IoRT Systems Based on Hybrid NTN Networks

This category of IoRT systems seeks to exploit the combined strengths of satellites and UAVs to supplement the satellite backbone infrastructure with dynamic and mobile capabilities, enabling targeted coverage. However, this complexity translates into more sophisticated systems, resulting in fewer research works in this regard. Therefore, we identified a total of 11 related research works in this category, classified according to their core research objectives into the following subsections. As anticipated, the limited number of research works does not address the full range of research objectives identified in Section 4. Consequently, as we delve deeper into Section 8, a research gap is identified, emphasizing potential areas for future investigation.

### 6.1. Enhancement of Energy Efficiency

In [125], the authors investigate the feasibility of a satellite-aerial-terrestrial integrated network to effectively support the expanding IoT landscape. The study centers on cognitive radio-based networks, leveraging their capabilities to address the critical challenge of spectrum resource management in response to the increasing demands of IoT devices. The study emphasizes the significance of spectrum sharing across satellite, aerial, and terrestrial networks, highlighting the role of cognitive networks in fulfilling IoT requirements. The authors propose a cooperative beamforming scheme designed to optimize IoT communications’ security and energy efficiency under constrained energy resources.

Leveraging UAV relays, ref. [126] explores energy-efficient resource allocation in a two-hop uplink communication for Space-Air-Ground IoRT networks. In remote areas where ground base stations lack reach, satellites and smart devices rely on UAV relays for seamless communication. By optimizing sub-channel selection, uplink transmission power control, and UAV relay placement, the proposed iterative technique maximizes system energy efficiency, addressing the challenges of IoRT networks. The Lagrangian dual decomposition approach is employed to determine the optimal sub-channel selection and power control policy based on UAV relay deployment positions. Successive convex approximations are then used to determine UAV relay placement. Numerical results demonstrate that the proposed scheme significantly improves system energy efficiency.

In [127], the authors propose a novel UAV-LEO integrated data collection system that addresses the challenges posed by the network’s heterogeneity and the limited mobility of UAVs and LEOs. The system aims to maximize data gathering efficiency by optimizing IoT device bandwidth allocation and UAV trajectory optimization. Optimizing the transmission powers of UAVs and selecting LEO satellites are proposed to maximize data upload volume and minimize UAV energy consumption. Simultaneously optimizing IoT-UAV data collection and UAV-LEO data transmission is achieved by considering the relay role and cache capacity constraints of UAVs. This problem is efficiently solved using sequential convex approximation (SCA) and block coordinate descent (BCD). The proposed system is shown to perform efficiently in terms of energy consumption and total upload data volume.

Table 10 summarizes key research efforts in enhancing the energy efficiency of IoRT systems utilizing hybrid NTN architectures. Researchers have proposed various techniques, including cognitive network architectures, cooperative beamforming schemes, and iterative-based algorithms, to optimize energy consumption and maximize network performance under constrained energy resources. These advancements demonstrate the promising potential of hybrid NTN networks in addressing the energy efficiency challenges in IoRT applications.

### 6.2. Enhanced Throughput/Transmission Rate

In [128], the authors present a simulation of a scenario involving a large number of UAVs hovering in the sky and a GEO-deployed relay satellite. They delve into analyzing and optimizing the performance of IoRT networks with UAV access and GEO satellite backhaul. To address the modeling challenges posed by the complex two-tier network, they developed a two-level queue system composed of multiple UAV queues on the first level and the satellite queue on the second level. To design the two-level transmission service for each UAV group, they introduce the stochastic network calculus (SNC)-based min-plus convolution and the leftover service, which are used as mathematical tools to effectively address the complexities of the network structure. The simulation results demonstrate that the proposed approach is effective in maximizing throughput while guaranteeing delay bounds.

Satellite and UAV integrated networks have the potential to enable a variety of IoRT applications. With regard to IoRT applications, the authors of [64] investigate methods for enhancing downlink transmission. Radio frequency technology is used for satellite-to-UAV links, and free-space optical (FSO) technology is used for UAV-to-IoRT connectivity. To address the scarcity of statistical Channel State Information (CSI), the authors devise an optimization problem aimed at maximizing the system’s Ergodic Sum Rate (ESR) while adhering to power and IoRT device rate constraints. To address this nonconvex problem, a beamforming scheme based on the Alternating Direction Method of Multipliers (ADMM) is proposed. To reduce implementation complexity, a suboptimal zero-forcing approach is introduced. For the proposed BF schemes, closed-form ESR expressions are derived, and simulations are employed to validate the theoretical analysis.

Addressing the critical absence of 5G coverage in maritime regions, ref. [129] proposes a nearshore network leveraging on-shore terrestrial base stations and tethered UAVs in virtual clusters. Employing non-orthogonal multiple access for efficient data transmission to maritime IoT devices, the network forms a hybrid satellite-UAV-terrestrial architecture. To mitigate interference, a joint power allocation problem is formulated to maximize network sum rates. Large-scale channel state information extracted from maritime IoT device locations is employed to optimize system overhead. An iterative power allocation algorithm is introduced, demonstrating the potential for enhanced coverage in maritime on-demand networks based on NOMA.

Due to the limited transmission power of smart devices in IoRT networks, UAVs can be used as relays to transmit data from smart devices to LEO satellites in Space-Air-Ground (SAG)-IoRT systems. To maximize system capacity, the authors of [130] propose a joint optimization approach that considers smart device connection scheduling, power control, and UAV trajectory. To achieve maximum system capacity, researchers alternately iterate smart device connection scheduling, power control, and UAV trajectory design. The proposed iterative solution outperforms both the static UAV scheme and the dynamic UAV scheme with a circular trajectory.

Table 11 summarizes research on IoRT systems based on Hybrid NTN Networks to enhance throughput and transmission rate. These studies propose novel frameworks and resource allocation strategies to address heterogeneity challenges and maximize network performance.

### 6.3. Enhanced and Timely Data Acquisition

The study in [69] explores a satellite-based 5G scenario for massive machine-type communication, utilizing an intermediate layer of UAVs for efficient data collection from sensors dispersed across vast rural areas. Specifically, the application focuses on the timely detection of fire alarms. In the scenario under study, terrestrial internet connectivity is limited or unavailable, making UAVs the ideal solution for gathering sensor data and relaying it to a control station via satellite. The study proposes a system architecture tailored to this scenario and develops an analytical model to characterize the average delay involved in transmitting fire alarm notifications from the field to the control center. The mobile gateway framework provides an alternative for collecting data while also reducing the problem of network partition caused by static gateways. To validate the proposed approach, simulations were conducted, demonstrating its effectiveness.

To optimize data acquisition strategies in the IoRT, a novel integrated space–air–ground network employing unmanned aerial vehicles and low-earth orbit satellites is proposed [65]. The authors differentiate between delay-sensitive and delay-tolerant data, tailoring transmission methods accordingly. For delay-sensitive data, a real-time relay via LEO satellites ensures timely delivery, while delay-tolerant data are stored on UAVs for subsequent transmission to a data center. To address the complex optimization problem, a multidimensional algorithm, Rate Demand-Based Joint Optimization (RDJO), is introduced. RDJO optimizes UAV deployment, IoRT device bandwidth allocation, and UAV transmission power, significantly enhancing IoRT data acquisition efficiency. Simulations comparing RDJO to conventional algorithms validate its efficiency.

The research in [131] investigates the combined use of UAVs and LEO satellite networks to provide low-latency access to IoRT sensors. The authors evaluate LEO satellite-assisted UAV data collection, which utilizes two communication mechanisms: delay-tolerant data are transmitted to Earth via the carry-store mode of UAVs, while delay-sensitive data are transmitted via the UAV satellite network. Considering the limited payloads of UAVs, the authors aim to minimize the total energy cost of the UAV trajectory and transmission while meeting IoRT requirements. The efficiency of the proposed approach is proven by numerical results.

Table 12 summarizes the work on IoRT systems based on hybrid NTN networks for enhanced and timely data acquisition. Bandwidth allocation and UAV placement strategies are employed to optimize data transmission delay under delay sensitivity constraints.

### 6.4. Enhancement of Edge Computing Capabilities

The authors in [132] propose an MEC-enabled NTN for wide-area, time-sensitive IoT scenarios in remote regions. The proposed system utilizes a hierarchical architecture comprising GEO satellites, UAVs, HAPs, and LAPs, with a focus on a UAV swarm equipped with an MEC server and a satellite that relays data to the cloud server via a gateway. The NTN employs a cell-free architecture to address the complex propagation environment between communication and MEC and simultaneously coordinates resources to accommodate on-demand coverage and the uneven distribution of IoT devices. The problem is modeled using large-scale CSI to minimize computing latency in the NTN, while a power allocation and data stream scheduling system is also introduced.

Table 13 summarizes the research work on IoRT systems based on hybrid NTN networks for enhancement of edge computing capabilities. MEC-enabled framework along with large-scale CSI are employed to optimize latency and coverage for IoT devices.

## 7. IoRT Systems Based on Aerial NTN Networks

The reliance on UAVs as the sole means of connectivity for isolated areas not covered by terrestrial wireless networks presents obvious limitations. As a result of these limitations, we identified a total of four research works in this category, classified according to their core research objectives into the following subsections. As expected, the limited number of research works covers only a subset of the research objectives identified in Section 4. Consequently, a research gap is evident, indicating potential areas that can be explored in future studies.

### 7.1. Enhancement of Energy Efficiency

This work introduces a drone-powered IoT relay system for efficient data collection in remote environmental monitoring applications [133]. The system utilizes 5-GHz communication technology for high-speed data transmission and low-power LoRa technology for energy optimization, enabling a sustained throughput of 3.5 MB/s for cached data collection at an altitude of 140 meters. This innovative approach offers a promising solution for intelligent environmental monitoring in regions lacking public networks, significantly enhancing data transmission from critical areas.

In this study, the authors examine the future of 6G wireless communication networks, seeking improved metrics to connect a large number of devices, which presents challenges for current IoT applications [134]. Integrating non-orthogonal multiple access and spatial modulation techniques, a novel method called Spatial NOMA (S-NOMA) is introduced to increase energy efficiency. Multiple-Input Multiple-Output (MIMO) enables spatial modulation to selectively activate transmission antennas per symbol interval, thereby optimizing data rates and minimizing interantenna interference. In addition, an energy efficiency-focused optimization method for power allocation is proposed for the S-NOMA scheme. This method improves energy efficiency for all users by incorporating antenna selection bits from all users, in contrast to traditional NOMA. Simulation results demonstrate the improved performance of S-NOMA with energy-efficient power allocation over conventional NOMA.

Table 14 summarizes the work on enhancing the energy efficiency of IoRT systems using aerial NTN Networks.

### 7.2. Enhanced Throughput/Transmission Rate

As a solution for the 6G heterogeneous IoT, Clustered Non-Orthogonal Multiple Access (C-NOMA) is proposed in [135] in order to address spectrum efficiency, system throughput, and receiving-end complexity. It uses UAVs as Low-Altitude Platform Stations for localized communication reinforcement and high-altitude platform stations for broader coverage. The study aims to optimize four key factors: spectrum allocation, power control, horizontal coordinates, and hovering altitude for UAV 3D location planning and resource allocation. Simulations and theoretical analysis demonstrate that the proposed two-stage solution substantially improves the system uplink sum rate with relatively low complexity compared to previous methods.

The study in [136] proposes a Clustered NOMA (C-NOMA) system that utilizes UAVs as aerial base stations for Wireless-Powered Communication (WPC) to maximize the uplink average achievable sum rate of IoT terminals [136]. WPC enables IoT terminals to harvest energy from downlink radio frequency signals and subsequently use the harvested energy to transmit data to the uplink. By optimizing UAV trajectory and subslot allocation in a synergistic manner, the proposed system ensures that the uplink sum rate and UAV mobility constraints are met. The complex and nonconvex optimization problem is efficiently solved using an iterative algorithm. Numerical results validate the effectiveness of this scheme for a UAV-supported C-NOMA system.

Table 15 presents a concise summary of the information presented in this section.

## 8. Recent Trends, Observations, and Future Research Challenges

This section analyzes current research trends observed in Section 5, Section 6 and Section 7 and identifies future research challenges related to integrating NTNs into the IoRT environment.

### 8.1. Research Trends and Observations

IoRT presents unique challenges for connectivity and data gathering and processing, demanding innovative solutions that are cost-effective, scalable, and efficient. To address these challenges, we have identified key research trends that are shaping the future of IoRT. This subsection delves into promising technologies like CubeSats, NOMA, machine learning, and MEC. Examining these technologies provides valuable insights into how IoRT can overcome its hurdles and realize its full potential for revolutionizing remote IoT applications.

- *CubeSats: A Cost-Effective and Scalable Solution for Remote IoT Connectivity*
  Miniaturized satellites known as CubeSats are rapidly gaining traction as a cost-optimized solution for establishing communication links with geographically dispersed IoRT systems. Their miniature size and inherently low energy requirements make them ideal for constructing network constellations tailored to specific mission profiles and optimized for minimized energy expenditure. Furthermore, CubeSats exhibit exceptional scalability and adaptability, enabling the deployment of dense constellations with extensive coverage areas, particularly in regions beyond the reach of terrestrial infrastructure. This capability is further augmented by their proficiency in forming dynamic collaborative networks, which maximizes resource utilization and flexibility. Furthermore, CubeSats play a crucial role as excellent research platforms, promoting progress in compact electronics, energy-conserving techniques, and cost-efficient deployment strategies. These developments will have an immediate effect on the connection of IoRT systems because they lay the groundwork for future global connectivity that will be both more affordable and more accessible.
- *Softwarization Driving Agile and Scalable Remote IoT Networks*
  A key research path for NTN-based IoRT systems is centered around the softwarization of network infrastructure. The integration of SDN and NFV offers a flexible and dynamic architecture to achieve cost-efficient deployment in NTNs. Researchers are exploring the potential of software-defined radio for cost-effective gateway implementation, addressing interoperability challenges, and enhancing adaptability in diverse network environments. Additionally, the adoption of SLA-based automatic network slicing frameworks can also be noted as an emerging trend, enabling the efficient allocation of resources and customization of network slices tailored to the specific requirements of IoT devices. These trends collectively signify a shift towards more agile, cost-effective, and scalable solutions for the evolving landscape of remote IoT-based NTNs.
- *NOMA: A Key Enabler for Scalable and Efficient IoRT Connectivity*
  NOMA enhances spectral efficiency by enabling more devices to share the available frequency spectrum, thereby increasing capacity. This directly translates to increased network capacity, facilitating enhanced connectivity for remote IoT devices communicating with satellites and UAVs. NOMA, by leveraging a dynamic scheduling scheme, can dynamically adjust resource allocation based on the varying needs of IoT devices. This allows the system to handle a mix of devices with different data rates, latency sensitivities, and QoS demands by dynamically adapting the transmission parameters for each device. This adaptability further mitigates latency issues by prioritizing critical information transmission for real-time applications. NOMA’s inherent scalability ensures effective connectivity in dynamic environments, accommodating a growing number of devices and users. Moreover, its compatibility with emerging technologies like edge computing and machine learning further enhances performance and facilitates efficient resource management and optimization.
- *Machine learning-enabled IoRT: Optimizing Network Performance*
  Machine learning is revolutionizing IoRT by unlocking the full potential of NTN systems. ML algorithms excel at optimizing resource allocation within the NTN network and for its users. They achieve this through efficient bandwidth distribution via techniques like predictive demand forecasting, dynamic traffic routing, and priority-based scheduling. Furthermore, reinforcement learning and deep RL can be effectively harnessed to mitigate the Doppler effect in satellite communication. These techniques accurately predict frequency changes and promptly adapt modulation techniques, leading to improved signal quality and data transmission. Beyond network optimization, ML plays a crucial role in data management. It helps identify anomalies, reduce data size, and extract relevant features, enabling efficient analysis and informed decision-making. This empowers the use of NTN for the IoRT, providing robust and effective connectivity solutions for diverse IoRT challenges.
- *MEC-enabled IoRT Task Offloading*
  Integrating MEC architecture with NTNs holds significant potential to enhance the performance of NTN-based IoRT systems. Depending on the NTN architecture, this integration can be achieved by incorporating computing capabilities into satellites or UAVs, essentially forming edge servers. These edge servers, positioned closer to IoT devices, can offload IoRT processing tasks, reducing reliance on distant cloud servers. Utilizing coordination techniques, often based on ML, enables the dynamic allocation of offloaded tasks to achieve various objectives, such as energy-efficient channel allocation, maximizing the number of offloaded tasks, or minimizing latency through delay-aware task offloading. Furthermore, real-time collaboration may enable edge nodes to exchange processed data and make collective decisions, further improving the effectiveness of MEC.

### 8.2. Future Research Challenges

Research on NTN-based IoRT systems continuously seeks to improve existing techniques, and new approaches and technologies offer further potential for advancement across all design objectives. However, despite significant research dedicated to IoRT systems based on space-borne NTN networks and their alignment with the design objectives outlined in Section 4, a notable gap exists for IoRT systems based on Hybrid and Aerial NTN networks. Research in these domains has not comprehensively addressed all identified design objectives, as will be further explored in the following.

- *Enhancing Coverage, Data Transmission Reliability and Timely Data Acquisition*
  For objectives related to coverage, data transmission reliability, and timely data acquisition in IoRT systems based on Hybrid and Aerial NTN networks, the identified research gap is partially attributed to the reliance on the mobility capabilities of UAVs, particularly LAPs, which can offer localized coverage, potentially improving both reliability and coverage and facilitating timely data acquisition. However, it is also crucial to acknowledge their inherent limitations in range, energy consumption, and integration with existing infrastructure. Dedicated research efforts are essential in these areas to develop effective schemes that comprehensively address all design objectives, unlocking the full potential of UAVs and bridging the gap towards robust and reliable IoRT networks.
- *Addressing Cost-Effective Deployment Challenges*
  Furthermore, a critical research gap persists in the area of cost-effective deployment and operation of IoRT systems based on Hybrid and Aerial NTN networks. Especially in the case of hybrid NTN networks, their inherently more complex architecture is expected to pose more challenges. Consequently, this presents fertile ground for exploration, where innovative approaches like CubeSats, which are explored for space-borne NTN networks, can be applied. However, it is important to note that while leveraging research from space-borne NTN networks to hybrid and aerial networks and vice versa can be promising, direct technology transfer is often not feasible. The research community needs to carefully assess the applicability of existing knowledge in each specific case, considering the distinct characteristics and challenges of each network type.
- *Enhancing Edge Computing Capabilities*
  Another significant research opportunity lies in enhancing the edge computing capabilities of IoRT systems based on aerial NTN networks. Exploring and unlocking the potential synergies between UAV swarms, edge computing, and AI holds significant promise for revolutionizing IoRT systems based on aerial NTN architectures. By fostering deeper research and development in this domain, we can pave the way for a future with intelligent, distributed networks that seamlessly connect and empower a vast array of remote devices. This holds the potential to revolutionize fields ranging from environmental monitoring and disaster response to precision agriculture.
- *Energy Efficiency of IoRT Systems*
  While enhancing edge computing capabilities presents a transformative opportunity for IoRT systems, another equally important research area lies in ensuring the energy efficiency of these networks. Although the research community has made efforts to address this challenge, further work remains to be conducted. Moreover, the ongoing drive to improve energy efficiency in NTNs, particularly for seamless integration with remote IoT systems, is expected to be incorporated into the upcoming 3GPP standards. This is crucial for the sustainability of NTNs, and therefore, the implementation of technologies like extended Discontinuous Reception (eDRX) and the adoption of energy-as-a-service criteria are expected to have a significant impact on the development of energy-efficient models for future NTNs, aligning with the recommendations outlined in the 3GPP standards.

## 9. Future Outlook toward 6G-Era

Despite the transformative impact of 5G networks on user experiences and IoT applications, their limitations, particularly in remote or isolated regions, are undeniable. These areas lack terrestrial network coverage, hindering seamless access and service provision for 5G-enabled IoT applications. In response to this challenge, ongoing research and scientific trends highlight the need for continuous 5G standard evolution, particularly in the realm of non-terrestrial networks. By seamlessly integrating these networks with their terrestrial counterparts, we pave the way for the emergence of futuristic 6G networks [137].

This shift in focus aims to address the limitations of current IoT connectivity and unlock new opportunities for remote applications [138,139]. As we look towards the 2030s, the advent of 6G networks is poised to overcome these obstacles, fulfilling the fundamental need for ubiquitous connectivity [140,141]. Research and industry are actively exploring the capabilities and requirements of 6G, with ultra-high frequency communications, artificial intelligence, edge computing, and non-terrestrial networks at the forefront [142,143,144]. These elements together will construct a fully networked 6G world, empowering remote IoT applications and emerging services. A 6G network provides the vision of a unified environment that seamlessly connects the globe, bridging the divide between remote and accessible areas [145,146,147].

### 9.1. NTNs Vision and The Forthcoming Energy-Awareness

**Figure 4:**
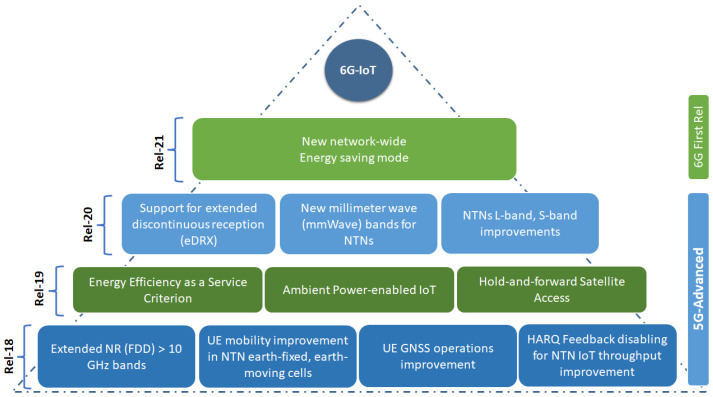
Overview of planned 3GPP standard releases on NTN.

Figure 4 illustrates the various stages of 5G network upgrades towards 6G-IoT as per the 3GPP Release cycles. Anticipating additional technical advancements, the evolution from 5G to 5G-Advanced standards is expected to address the limitations of non-terrestrial networks, particularly in areas with no terrestrial coverage. The ongoing development of 5G technology aims to enhance performance and cater to emerging use cases.

The inherent versatility of 5G lays a robust foundation for enabling NTNs. Given the complexity of NTNs, especially satellite communication networks, a holistic approach is necessary for their design. Across multiple 3GPP releases, considerable attention has been dedicated to NTN design [72], signaling a commitment to making 5G from space a reality. Activities beyond standards are deemed essential for integrating non-terrestrial networks with IoT [41]. Subsequent 3GPP upgrades are poised to unlock possibilities for servicing remote locations.

### 9.2. NTNs in 3GPP Releases 18 and 19

Enhancements in 3GPP Release 18 (Rel-18) for NTNs encompass support for frequencies above 10 GHz, integration with 3GPP New Radio (NR), improved mobility management, handover procedures, power-saving features, and enhanced security and reliability [12,148]. These improvements are projected to facilitate diverse use cases, including global broadband connectivity, support for remote and underserved areas, disaster relief, connected vehicles, drones, and Industrial IoT. Furthermore, Rel-18 is expected to introduce the option to disable Hybrid Automatic Repeat Request (HARQ) feedback for NTN IoT by default, which can significantly improve throughput for devices with sporadic data traffic.

In the context of Release 19 (Rel-19), support for satellite-based 3GPP communication implies the availability of a backhaul network between the radio access node and the core network, as well as the wireless device’s ability to utilize existing Global Navigation Satellite System (GNSS) functionality [149]. For the Rel-19 discussion, 3GPP will explore scenarios involving discontinuous backhaul connections. For example, a satellite could regularly orbit Earth and receive data from a location without direct transmission. In this scenario, it is necessary for satellite communication to have hold-and-forward capabilities.

While devices may use a 3GPP-based satellite communication architecture to determine their location in certain scenarios, the primary driver behind the upcoming 3GPP specifications for NTNs is the development of direct links with both user equipment (UE) and IoT devices.

In addition, Rel-19 is projected to integrate support for ambient power-enabled remote IoT devices, enabling them to harvest energy from their surroundings to power their operations. Apart from this, the Hold-and-Forward (HF) feature, which is also expected and being studied in Rel-19, is designed to overcome the issues of latency and reliability that are commonly encountered in satellite communications as it enables the satellite to temporarily hold data packets prior to their transmission to the ground station. Consequently, the hold-and-forward technique plays a crucial role in guaranteeing the integrity of data in challenging and remote conditions by storing packets that could potentially be lost as a result of interference or obstacles.

The scientific community has consistently emphasized that adherence to the 3GPP standards for NTNs is crucial to the successful implementation of future enterprise strategies [150]. This recognition of NTN’s significance as a key work item and research area within the 3GPP has attracted a growing number of companies seeking to participate in this burgeoning field. However, the organization is actively exploring potential collaborations with emerging space companies and established corporate entities to harness the potential of 3GPP-based NTNs and expand their reach.

#### Energy Efficiency Challenges

In 3GPP Rel-18, significant focus has been directed towards enhancing the energy efficiency of NTNs [12]. These enhancements are intended to reduce power consumption in NTN base stations and user UEs while maintaining or even improving overall performance. One noteworthy improvement involves refining beamforming techniques, enabling more precise targeting of signals from NTN base stations towards UEs. This targeted signal delivery minimizes the power required for data transmission and reception. Furthermore, introducing new power-saving modes for NTN UEs is another crucial addition. These modes allow UEs to conserve power during periods of inactivity or when data transmission or reception is not actively in progress. In addition, network-assisted power management is incorporated to assist UEs in effectively managing power consumption. The network will provide valuable information about traffic conditions and available energy sources, empowering UEs to optimize their power usage efficiently [151].

Furthermore, leveraging AI and ML has emerged as a pivotal strategy for enhancing energy efficiency in NTNs. AI and ML algorithms are employed to dynamically adjust critical parameters such as beamforming, power levels, and network topology, leading to significant energy savings [152]. As recognized by 3GPP, energy efficiency will be a major focus of Rel-19, with its importance reflected in its consideration as a service criterion. Telecommunications operators are actively pursuing strategies to reduce energy usage in network infrastructure and wireless devices. Optimizing power consumption in the network equipment is paramount for achieving cost efficiency and reducing operating costs [153]. However, it is crucial to strike a balance between power reduction and maintaining QoS for end-users.

### 9.3. Future 3GPP Standard Releases

Future versions beyond 3GPP Rel-18 and Rel-19 pave the way for future technical upgrades of 5G-NTN on a physical level, as well as a reconfiguration of 5G networks by 2030, when the first version for 6G networks will be released. This direction necessitates substantial investigation and is open to scientific testing and simulations by the scientific community. The need for improvements in energy efficiency and the limited availability of spectrum require further enhancements.

To tackle these challenges, 3GPP Release 20 (Rel-20) initiatives will address them by introducing innovative technologies such as extended discontinuous reception (eDRX) and millimeter wave (mmWave) bands [150,154,155,156]. The eDRX is a power-saving technology that enables NTN devices, like satellites, to enter periodic low-power sleep mode while maintaining continuous network communication. This is crucial for satellites that have limited energy resources, as it helps to increase their operational lifespans and decrease expenses. 3GPP specifications for mmWave bands for NTNs introduce novel channel models, modulation and coding schemes, and beamforming techniques. These advancements are specifically designed to optimize the propagation characteristics and interference environment of NTNs. As a result, they provide improved bandwidth, reduced susceptibility to interference, and the potential for high data rates and low latency [155].

In the direction of the space bands, specific advancements were made for L-band and S-band in the context of NTNs. For L-band, the standardization included Single Carrier Modulation and Coding Schemes (SC-MCS), providing enhanced spectral efficiency and lower latency compared to conventional multi-carrier modulation. Additionally, new channel models and interference mitigation techniques tailored for L-band NTN links were introduced [157]. In the case of S-band, the focus was on optimizing Dual Polarized (DP) and Quadrature Polarized (QP) antenna systems, improving resource allocation, and modulation schemes. The standardization also included beamforming techniques to concentrate transmitted signals, thereby extending the capacity and range of S-band NTN links. These standardized improvements in the L-band and S-band, driven by 3GPP Rel-20, are poised to significantly enhance the capabilities of NTNs, fostering innovations in areas like maritime connectivity, aerial broadband, and disaster relief [157,158].

In conclusion, 3GPP Rel-20 and Release 21 (Rel-21) mark a pivotal transition towards the next generation of the Internet of Things, commonly referred to as 6G-IoT. These releases signify significant progress in Non-Terrestrial Networks, introducing millimeter wave (mmWave) bands, enhancing L-band and S-band, and, notably, introducing network-wide energy-saving modes. Rel-20’s mmWave bands provide expanded bandwidth, which is crucial for supporting emerging applications like video streaming, while advancements in L-band and S-band improve spectral efficiency and reduce latency, fostering wider coverage in remote areas. Building upon Rel-20, Rel-21 introduces energy-saving modes, enabling NTN devices to operate more sustainably.

The synergistic impact of these advancements positions 6G-IoT as a transformative force with the potential to revolutionize the connectivity and quality of experience (QoE) of users in remote areas and utilize services upon the remote IoT, including remote ultra-high precision agriculture, remote environmental condition monitoring, and remote supply chain optimization.

## 10. Conclusions and Future Work

In conclusion, this survey paper emphasizes the importance of non-terrestrial networks in the Internet of Remote Things ecosystem. These networks overcome the limitations of traditional terrestrial networks, enabling reliable connectivity in geographically isolated areas and challenging environments. The paper identifies seven key objectives for NTN research: energy efficiency, cost-effective deployment/operation, enhancement of coverage and availability, enhancement of throughput/transmission rate, enhancement of data transmission reliability, enhanced and timely data acquisition, and enhancement of edge computing capabilities. By addressing these objectives, NTNs for IoRT have the potential to revolutionize the way data are collected, processed, and utilized, enabling a wide range of applications in remote and isolated environments.

However, to fully unlock this potential, further research is necessary. Building upon the findings of this survey, our future work will focus on two key areas:

- Investigating the impact of distance, network resources, and aerial platform types on the feasibility and performance of remote IoT connectivity via NTNs. This will involve developing models to predict network performance and identify optimal configurations for different deployment scenarios.
- Exploring energy-efficient communication protocols and developing power-aware routing algorithms that leverage the unique characteristics of NTNs to minimize energy consumption. This may involve integrating renewable energy sources into remote IoT devices and quantifying the environmental impact of different IoRT deployment scenarios.

By addressing these research areas, we aim to contribute to the development of a sustainable and efficient IoRT ecosystem, unlocking the full potential of NTNs.

## Figures and Tables

**Table 1:** Internet of Remote Things (IoRT).

References	IoRT Environments	Description
[7,21,22]	Internet of Agricultural Things (IoAT)	Remotely monitor and manage agricultural operations, optimizing crop yields, reducing water usage, and enhancing pest control, thereby improving agricultural sustainability and profitability.
[23,24]	Internet of Maritime Things (IoMT)	Enhance marine security, optimize shipping routes, and reduce environmental impact by monitoring and tracking vessels, detecting illegal fishing activities, and optimizing fuel consumption.
[26,27,28]	Internet of Underground Things (IoUT)	Monitor and maintain underground infrastructure, including pipelines, tunnels, and power lines, to prevent leaks, collapses, and outages, ensuring public safety and infrastructure reliability.
[29,30,31,32]	Internet of Underwater Things (IoUwT)	Monitor and protect underwater ecosystems, detect pollution, and perform research on marine life by tracking fish migrations, assessing the health of coral reefs, and monitoring water quality.
[33,34]	Internet of Satellite/Space Things (IoST)	Enhance navigation and communication in space, improve network reliability, and enable remote monitoring of satellites by providing real-time data and alerts.
[35,36,37]	Internet of Battle Things (IoBT)	Enhance situational awareness and decision-making in combat conditions by providing real-time data on enemy movements, troop locations, and battlefield conditions.
[38]	Internet of Arctic Things (IoAT)	Support search and rescue operations, conduct scientific research on Arctic ecology, and monitor the impact of climate change in the Arctic by tracking and analyzing environmental data.

**Table 2:** Related surveys and reviews.

	Reference	Year	Main Focus	NTNs		Coverage of IoRT Connectivity Solutions	Coverage of Energy Efficiency Solutions
				Satellites	UAVs		
1	[39]	2015	Overview of satellite technologies for IoT Applications	✓	✕	Partially	✕
2	[40]	2017	Utilization of LEO satellites in IoT	Partially	✕	Partially	✕
3	[41]	2019	Utilization of satellite and UAV technologies in 5G-IoT networks	Partially	✓	Partially	✕
4	[42]	2020	AI-based techniques to improve the performance of NTN-based IIoT services	Partially	✓	Partially	Partially
5	[43]	2021	Hybrid Satellite–Terrestrial Networks (HSTNs)	✓	✓	Partially	Partially
6	[44]	2021	Satellite-based IoT solutions for remote/rural areas	✓	✕	Partially	✕
7	[45]	2021	Integration status of IoT and space communication technologies	✓	✓	Partially	Partially
8	[24]	2021	IoT Maritime Communication Networks (MCNs)	✓	✓	Partially	Partially
9	[46]	2022	UAV-enabled Wireless Sensor Networks (U-WSNs)	✕	✓	Partially	Partially
10	[47]	2022	Evolution of NTNs in 5G context	✓	✓	Partially	Partially
11	[48]	2022	Communication solutions for IoT applications in cellular, wide-area, and non-terrestrial networks	Partially	✓	Partially	Partially
12	[49]	2023	Space–air–ground–sea-integrated (SAGSI) networks deployment resource optimization analysis	✓	✓	Partially	Partially
13	[50]	2023	Satellite communications overview for multi-orbit IoT	✓	✕	Partially	✕
14	This Survey	2024	Non-terrestrial networks, including UAV and satellite technologies, for energy-efficient connectivity of remote IoT Devices	✓	✓	✓	✓

**Table 3:** IoRT systems based on space-borne NTN networks: **enhancement of energy efficiency**.

Ref.	NTN Architecture	Description
[73]	LEO	Energy optimization utilizing the repetition factor of data symbols on multiple subcarriers of the transmitted OFDM signal and the mean number of coded packets required to satisfy dynamic requirements.
[74]	LEO	A novel random access preamble design and detection mechanism for IoT devices in LEO satellite-based NTNs that addresses the energy-intensive nature of random access.
[75]	LEO	A method for synchronizing sleep and wake-up periods with satellite availability to substantially enhance the battery life of IoT-NTN devices.
[76]	LEO	Combination of uplink signals from multiple LEO satellites to improve SNR and reduce power consumption for energy-constrained terminals.
[77]	LEO	Novel MEC framework for terrestrial-satellite IoT accompanied by an energy-efficient computation offloading and resource allocation algorithm named E-CORA.
[78]	LEO	Development of DeepCA, a deep reinforcement learning-based method for dynamic and energy-efficient channel allocation.
[79]	Generic	Development of a novel power control algorithm for IoT terminals in satellite-based networks that optimizes user distribution and SINR demand.
[80]	Generic	Development of a scheduling algorithm for multi-connectivity in multi-orbit NTNs to maximize uplink data rates while minimizing user terminal energy consumption.
[81]	LEO	Development of NOMA-based beamforming algorithms for tackling massive access in B5G IoT networks and minimizing total power consumption for non-critical and critical IoT applications.
[82]	LEO	Development of the Deep Reinforcement Learning method (DRL-QoS-RA) for online joint resource allocation to solve the uplink channel allocation and power control problems for satellite IoT systems.

**Table 4:** IoRT systems based on space-borne NTN networks: **cost-efficient deployment/operation**.

Ref.	NTN Architecture	Description
[83]	LEO NB-IoT	By compensating for residual Doppler shifts, it minimizes the number of required satellites while maintaining continuous communication.
[84]	LEO LoRa	Investigates the potential of LoRa protocol variations for a reduction in the number of required satellites.
[85]	Generic	A CubeSat SDN NFV-based architecture is proposed to achieve cost-efficient deployment and operation, enhanced geographic coverage, and improved throughput.
[86]	Generic	Employing Software-Defined Radio (SDR) for cost-effective gateway implementation and resolving interoperability issues.
[87]	LEO-GEO	Development of an uplink scheduling algorithm that optimizes the utilization of uplink transmission resources in order to achieve a fair distribution of resources and significant cost reductions.
[88]	LEO	Application of CubeSat nanosatellites as APs to establish a cost and energy-efficient IoT/M2M network using erasure coding. Using massive numbers of nanosatellites, area coverage is extended.

**Table 5:** IoRT systems based on space-borne NTN networks: **enhanced coverage and availability**.

Ref.	NTN Architecture	Description
[89]	LEO	Development of a beam-hopping-based satellite system for IoT that can support extensive coverage with fewer transmitters and offers flexibility to cope with time-variant traffic distributions.
[90]	Generic	Development of solar-powered satellite access points to provide energy autonomy and reliable internet connectivity in areas with irregular electricity supply.
[91]	LEO	Study of satellite coverage variations due to latitude shift.
[92]	LEO-Cellular	Development of a novel gateway placement algorithm that considers channel conditions and service demand distribution to optimize network resource utilization and coverage.
[93]	LEO	Development of a modeling framework for IoT-over-satellite access systems that aims to maximize link availability while minimizing interference.
[94]	GEO	Development of a NOMA multibeam satellite IIoT architecture in Ka-band to extend coverage to devices located in remote areas.

**Table 6:** IoRT systems based on space-borne NTN networks: **enhanced throughput/transmission rate**.

Ref.	NTN Architecture	Description
[95]	GEO	Development of a spectrum management scheme for human-to-human and machine-to-machine communications to improve spectral efficiency, throughput and fairness index.
[96]	LEO NB-IoT	Development of a resource allocation scheme to mitigate the issue of high differential Doppler among terrestrial user channels.
[97]	Nano-satellites	Precise beamforming directed towards specific IoT devices enhances received signals while minimizing interference.
[98]	LEO	Development of a slotted random access protocol based on Walsh codes for a satellite IoT scenario with sink nodes that aims to improve the throughput of the system, especially under high load.
[99]	GEO	Development of a geographical non-orthogonal multiple access-based multiuser beamforming (NOMA-BF) scheme to improve spectral efficiency in multi-beam satellite-based IoT systems.
[100]	LEO	Scheme that combines NOMA and RA technologies for asynchronous transmissions to achieve better throughput performance and an enhanced data transmission rate.
[101]	LEO	Development of a robust multigroup multicast beamforming scheme to improve spectrum efficiency and adequately serve a large number of IoT devices.
[102]	LEO NB-IoT	Development of an uplink scheduling technique for an LEO satellite-based mMTC-NB-IoT system that mitigates the differential Doppler shift, thereby improving the system’s performance in terms of throughput and block error rate.

**Table 7:** IoRT systems based on space-borne NTN networks: **enhanced data transmission reliability**.

Refs.	NTN Architecture	Description
[103]	LEO	Mathematical derivation of an electromagnetic Power Flux Density (PFD) mask for mitigating satellite interference on terrestrial communication systems in the same frequency band.
[104,105]	GEO	Development of a novel modulation and signaling scheme based on Chirp-Spread Spectrum (CSS).
[106]	LEO-GEO	Development of a reinforcement learning framework to optimize resource allocation and enable robust data transmissions.
[107]	Generic	Use of Network Coding to enhance the feedback mechanism of the HARQ process.
[108]	LEO-LoRA	A system architecture is proposed, along with the development of a data encoding and packaging scheme for the reliable transmission of IoT data over low-speed satellite links.
[109]	Generic	Development of a novel collision detection scheme for satellite-based IoT that enables rapid collision detection and load estimation while being robust to non-orthogonal interference.
[110]	Generic	Use of data compression and transmission optimization to improve the HARQ process as well as the timeliness of data.
[111]	LEO	Development of a novel access scheme for terrestrial-satellite IoT communication that maintains low access delay, robustness to channel impairments, and enables reliable data transmission.
[112]	LEO	Development of a robust beamforming scheme for NOMA-based integrated satellite-terrestrial IoT networks to minimize power consumption and ensure reliable communication for a large number of IoT devices.

**Table 8:** IoRT systems based on space-borne NTN networks: **enhanced and timely data acquisition**.

Ref.	NTN Architecture	Description
[113]	Generic	Development of an SLA-based automatic network slicing framework for ultra-dense SDN-based CubeSat networks.
[38]	LEO	Evaluating the feasibility of small-satellite swarms for IoT connectivity, focusing on reducing communication overhead and latency.
[67]	LEO	A study that demonstrates the feasibility of a satellite-enabled IoRT network for data retrieval in challenging Tibetan Plateau environments.
[114]	Generic	A metric is proposed to determine the optimal allocation of channel slots for data updates based on the timeliness of the information.
[115]	GEO	Development of a new packet scheduling algorithm for mixed traffic that improves throughput, ensures fairness, and maintains QoS for both delay-sensitive and delay-tolerant IoRT services.
[116]	Generic	Comparative performance evaluation of CoAP and MQTT protocols for rapid data delivery on satellite random access channels compliant with the DVB-RCS2 standard.
[117]	LEO	Development of a QoE-aware satellite constellation design scheme to enhance user experience in satellite IoT networks.
[118]	Generic	Development of a cooperative resource allocation mechanism that utilizes link condition feedback and adaptive coding to dynamically allocate resources and ensure consistent and reliable video streams.
[119]	LEO	Development of a joint optimization approach for successfully managing both data queuing delay and power consumption at the downlink of a NOMA-based satellite IoT system.

**Table 9:** IoRT systems based on space-borne NTN networks: **enhancement of edge computing capabilities**.

Ref.	NTN Architecture	Description
[120]	LEO	Development of a collaborative task processing scheme that leverages multiple satellites to process offloaded IoRT tasks.
[121]	LEO	Development of a hardware and software architecture that utilizes virtualization and flexible scheduling to transform traditional satellites into space edge computing nodes.
[122]	LEO	Modeling computation offloading as a Markov decision process and optimizing it using deep reinforcement learning to maximize offloaded tasks while ensuring delay performance.
[123]	LEO	Development of a policy optimization reinforced learning algorithm, which supports delay-sensitive task offloading to achieve better data transmission rates and reliability.
[124]	LEO	A hybrid computation offloading architecture is proposed, consisting of LEO satellites and cloud servers, to minimize task delay and reduce energy consumption utilizing multi-agent reinforcement learning.

**Table 10:** IoRT systems based on hybrid NTN networks: **enhancement of energy efficiency**.

Ref.	NTN Architecture	Description
[125]	LEO-UAV	A cognitive network architecture together with a cooperative beamforming scheme aiming to optimize energy efficiency under constrained energy resources.
[126]	LEO-UAV	Iterative-based algorithms are proposed to maximize systems’ energy efficiency in two-hop link communication for hybrid NTN-based IoRT networks.
[127]	LEO-UAV	Effective techniques have been developed to optimize UAV trajectories and transmit powers, aiming to maximize data upload volume while minimizing UAV energy consumption.

**Table 11:** IoRT systems based on hybrid NTN networks: **enhanced throughput/transmission rate**.

Ref.	NTN Architecture	Description
[128]	GEO-UAV	Development of a framework for analyzing and optimizing hybrid UAV-GEO networks, addressing heterogeneity challenges and maximizing throughput while guaranteeing delay bounds.
[64]	GEO-UAV	Development of a beamforming scheme based on the alternating direction method of multipliers to maximize the ergodic sum rate of the system.
[129]	Generic Satellite-UAV	A study of a hybrid satellite-UAV-terrestrial network architecture for maritime on-demand coverage that employs NOMA to manage interference and maximize network sum rate.
[130]	LEO-UAV	Development of a resource allocation scheme that maximizes system capacity by jointly optimizing connection scheduling, power control, and UAV trajectory.

**Table 12:** IoRT systems based on hybrid NTN networks: **enhanced and timely data acquisition**.

Ref.	NTN Architecture	Description
[69]	LEO-UAV	A novel system architecture is proposed, along with an analytical model to quantify the mean delay in alarm notifications, with a particular emphasis on ensuring timely alarm detection.
[65]	LEO-UAV	Joint optimization of bandwidth allocation for IoRT devices and UAV location deployment and transmission power while considering data delay sensitivity.
[131]	LEO-UAV	Combined use of UAVs and LEO satellite networks to delay aware access to IoRT sensors while minimizing the overall energy cost of UAVs.

**Table 13:** IoRT systems based on hybrid NTN networks: **enhancement of edge computing capabilities**.

Ref.	NTN Architecture	Description
[132]	GEO	Development of a process-oriented framework that integrates NTNs with MEC to enable wide-area, time-sensitive IoT applications. By utilizing large-scale CSI, it optimizes latency while ensuring on-demand coverage for IoT devices.

**Table 14:** IoRT systems based on aerial NTN networks: **enhancement of energy efficiency**.

Ref.	NTN Architecture	Description
[133]	LAP-UAV	Development of a low-cost, UAV-enabled LoRa IoT relay system for enhanced energy efficiency and high-speed environmental data acquisition.
[134]	UAV	Development of a spatial NOMA scheme combined with multiple access and spatial modulation techniques to enhance energy efficiency in 6G wireless networks for massive IoT applications.

**Table 15:** IoRT systems based on aerial NTN networks: **enhanced throughput/transmission rate**.

Ref.	NTN Architecture	Description
[135]	HAP-UAV	A 6G clustered-NOMA HAP-UAV system architecture is proposed, and a UAV 3D location planning and resource allocation scheme is developed that enhances system throughput.
[136]	LAP UAV	A trajectory optimization and subslot allocation scheme is proposed for UAV-based clustered NOMA wireless-powered communication systems to improve the uplink average sum rate for IoT terminals.

## Data Availability

Data sharing not applicable.

## Abbreviations

The following abbreviations are used in this manuscript:

3GPP	3rd Generation Partnership Project
AI	Artificial Intelligence
AoI	Age of Information
B5G	Beyond-Fifth-Generation
BR-MP	Bernoulli-Rician Message Passing
CoAP	Constrained Application Protocol
CSI	Channel State Information
CSS	Chirp-Spread Spectrum
DL	Deep Learning
DRL	Deep Reinforcement Learning
DVB-RCS2	Digital Video Broadcasting—Return Channel via Satellite 2
eDRX	extended Discontinuous Reception
EM	Expectation-Maximization
FDD	Frequency-division Duplex
FL	Federated Learning
GEO	Geostationary Earth Orbit
GF-RA	Grant-Free Random Access
GNSS	Global Navigation Satellite System
HAPs	High-altitude platforms
HARQ	Hybrid Automatic Repeat Request
HSTN	Hybrid Satellite-Terrestrial Network
IIoT	Industrial Internet of Things
IMDs	IoT Mobile Devices
IoAT	Internet of Agricultural Things
IoBT	Internet of Battle Things
IoMT	Internet of Maritime Things
IoRT	Internet of Remote Things
IoST	Internet of Satellite/Space Things
IoT	Internet of Things
IoUT	Internet of Underground Things
IoUwT	Internet of Underwater Things
IPF	Iterative Penalty Function
LAPs	Low-altitude platforms
LEO	Low Earth Orbit
LPWAN	Low-Power Wide-Area Network
LTE-M	Long-Term Evolution Machine Type Communication
mMTC	Massive Machine Type Communication
MEC	Mobile Edge Computing
MEO	Medium Earth Orbit
ML	Machine Learning
MLTS	Multi-Layer Tabu Search
MQTT	Message Queuing Telemetry Transport
MTC	Machine Type Communication
NB-IoT	NarrowBand Internet of Things
NC	Network Coding
NOMA	Non-Orthogonal Multiple Access
NTN	Non-Terrestrial Network
PFD	Power Flux Density
PPP	Poisson Point Process
PSO	Particle Swarm Optimization
QoE	Quality of Experience
QoS	Quality of Service
RL	Reinforcement Learning
SBD	Short Burst Data
SGP4	Simplified General Perturbation 4
SIC	Successive Interference Cancellation
SINR	Signal-to-Interference-plus-Noise Ratio
S-IoT	Satellite Internet of Things
SLA	Service Level Agreement
SNR	Signal-to-Noise Ratio
STI	Spatially Temporally Correlative Mutual Information
UAV	Unmanned Aerial Vehicle
UCSS	Unipolar-coded CSS
UE	User Equipment
URLLC	Ultra-Reliable Low-Latency Communications
VLEO	Very Low Earth Orbit
WDP	Winner Determination Problem
WSN	Wireless Sensor Network
